# The RNA promoter for pathogenic orthoflaviviruses replication is universal and serves as target for viral inhibition

**DOI:** 10.1371/journal.ppat.1014233

**Published:** 2026-05-18

**Authors:** Santiago Oviedo-Rouco, Lautaro Bertoni, Evelyn Mikkelsen, Carolina Sarto, Maria M. Gonzalez Lopez Ledesma, Horacio M. Pallarés, Claudia V. Filomatori, Antonia Bruce, Amanda E. Hargrove, Mehrnoosh Arrar, Andrea V. Gamarnik

**Affiliations:** 1 Fundación Instituto Leloir-CONICET, Ciudad Autónoma de Buenos Aires, Argentina; 2 Instituto de Cálculo, Universidad de Buenos Aires and CONICET, Ciudad Autónoma de Buenos Aires, Argentina; 3 Instituto de Química y Fisicoquímica Biológicas, Universidad de Buenos Aires and CONICET, Ciudad Autónoma de Buenos Aires, Argentina; 4 Department of Chemistry, University of Toronto, Mississauga, Ontario, Canada; Stanford University, UNITED STATES OF AMERICA

## Abstract

Orthoflaviviruses comprise a diverse genus of positive-strand RNA viruses that includes major human pathogens such as dengue, yellow fever, Zika, Japanese encephalitis, tick-borne encephalitis, and West Nile viruses. Despite their global impact, the molecular constraints that preserve viral replication across distinct vectors and host environments remain incompletely understood. Viral RNA replication depends on Stem-Loop A (SLA), a structured RNA element located at the 5′ end of the genome that recruits the viral polymerase NS5. Here, we examine the structural diversity of this RNA promoter across orthoflaviviruses. Using infectious clones, reporter viruses, and computational structural analyses, we show that SLAs from diverse mosquito- and tick-borne viruses are functionally interchangeable in the context of dengue and Zika virus infectious clones. Structure-function analyses reveal that conserved nucleotide contacts between the SLA top loop and the NS5 polymerase domain form a conserved interaction interface maintained across orthoflaviviruses, including insect-specific viruses. In contrast, other SLA sub-elements, such as the three-way junction and side stem, have diverged in a group-specific manner, yet co-evolution in mosquito and tick-borne viruses preserves the three-dimensional architecture and NS5 binding competence for viral replication. Guided by this conservation, we identify small molecules that bind SLA and inhibit replication across multiple pathogenic orthoflaviviruses. These findings uncover fundamental principles governing viral RNA promoter evolution and establish conserved RNA structures as promising targets for viral control.

## Author summary

Orthoflaviviruses are important human pathogens that cause recurrent outbreaks worldwide. These viruses infect both vertebrate and invertebrate hosts, including dengue, Zika, West Nile, yellow fever, tick-borne encephalitis, and Japanese encephalitis viruses. Their RNA genome encodes for viral proteins but also folds into structures that control key steps of infection. In this study, we focus on an RNA structure called Stem-Loop A (SLA), which acts as a promoter to initiate viral genome replication by recruiting the viral polymerase. Using molecular virology and computational structural analysis, we show that despite the diversity of orthoflaviviruses, the SLA is remarkably conserved in function. By swapping SLAs between different viruses, we demonstrate that these elements can substitute for each other, allowing viral replication. This indicates that a common structural mechanism underlies in the polymerase recognition and RNA synthesis across these viruses. At the same time, some regions of SLA vary between virus groups, reflecting adaptation to different hosts and transmission cycles. Importantly, we identify small molecules that bind to SLA and inhibit infections across multiple viruses. These findings reveal a widely conserved function of a viral RNA element and highlight it as promising target for the development of broad antivirals.

## Introduction

Orthoflaviviruses are a diverse group of enveloped, positive-sense single-stranded RNA viruses that include major emerging and reemerging human pathogens. Among them, dengue virus (DENV), West Nile virus (WNV), Zika virus (ZIKV), tick-borne encephalitis virus (TBEV), and yellow fever virus (YFV) drive recurrent epidemics with substantial morbidity and mortality [1,2]. Of significant concern, outbreaks of other less well-characterized orthoflaviviruses have been reported in humans and animals in different regions of the world [3]. Dengue, the most prevalent arthropod-borne viral disease, infects an estimated 400 million humans each year; more than a quarter of the world’s population lives in areas where DENV is now endemic [4]. In 2024, unprecedented epidemics of DENV swept the Americas, with outbreaks expanding to countries without dengue history [5]. ZIKV showed its epidemic potential during the 2015–2017 outbreaks in the Americas, when infection was linked to congenital malformations, including microcephaly, and neurological complications [6]. YFV is associated with high mortality and has reemerged in South America despite the availability of an effective vaccine [7].

**Fig 1:**
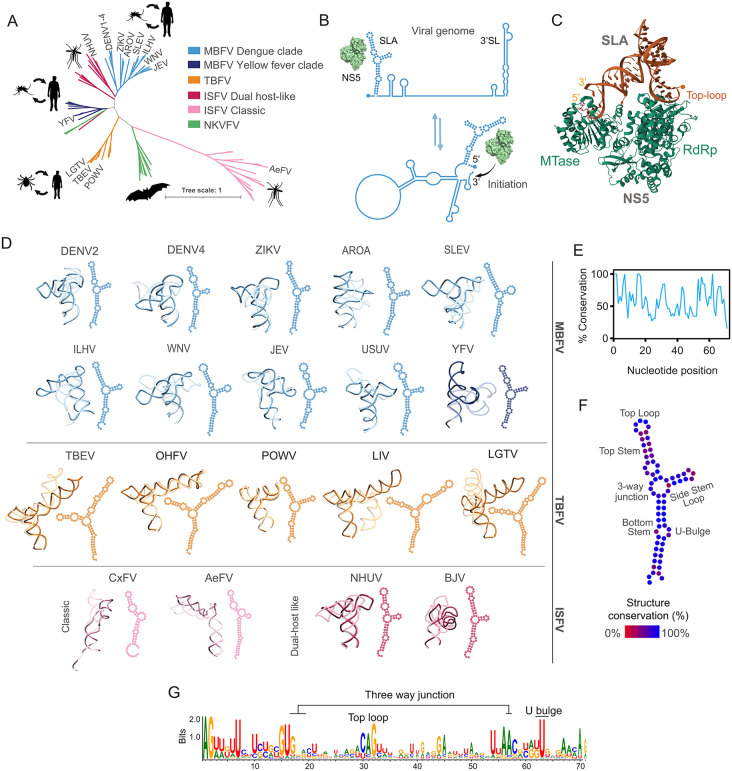
SLA conservation among orthoflaviviruses. **A.** Phylogenetic tree of orthoflavivirus built from a multiple sequence alignment of the NS5 sequence. Different ecological groups are indicated with different colors. **B.** Schematic representation of the mechanism of initiation of DENV negative strand RNA synthesis [31]. The SLA and 3’SL are indicated. Both, linear and circular forms of the genome are shown. The RdRp domain of NS5 initiates RNA synthesis at the 3’end of the circularized genome. **C.** Cartoon representation of the SLA-NS5 ribonucleoprotein complex determined by Cryo-EM, showing the two contact points: the SLA top loop with the RDRP and the 5’ end of the RNA with the MTase of NS5. SLA in orange and NS5 green. Protein data bank ID 8gzp. **D.** 2D and 3D models for selected SLAs corresponding to MBFV, TBFV and ISFV, as indicated on the right. Two models for each sequence are shown in opaque and translucent ribbons to highlight the variability among SLA conformations. **E.** Nucleotide conservation as a function of position calculated from the SLA sequence alignment. **F.** Graphical representation of the structural conservation of each nucleotide from a sequence-structure alignment. Color code from 0 to 100% is indicated. **G.** Sequence logo showing nucleotide conservation obtained from the SLA sequence-structure alignment built using sequences from every available orthoflavivirus. For clarity, numbering was adjusted to the DENV genome. Drawings used in this figure were from public domain clip-art images taken from NIH Bio Art. https://bioart.niaid.nih.gov/bioart/162, https://bioart.niaid.nih.gov/bioart/11, https://bioart.niaid.nih.gov/bioart/247, https://bioart.niaid.nih.gov/bioart/49, person standing modified from: https://bioart.niaid.nih.gov/bioart/555.

These viruses infect a broad range of vertebrate and invertebrate hosts and are ecologically classified according to their transmission cycle into mosquito-borne (MBFV), tick-borne (TBFV), insect-specific (ISFV), and flaviviruses with no known vector (NKVFV) (Fig 1A). Viruses associated with human disease are predominantly transmitted by mosquitoes or ticks. Considerable efforts have been devoted to the development of vaccines against several members of the genus, leading to the approval and deployment of vaccines against YFV, Japanese encephalitis virus (JEV), TBEV, and more recently DENV [8–12].These vaccines are expected to play a key role in disease control.

In parallel, extensive drug-discovery has focused on viral proteins. Despite these efforts, no specific antiviral drugs have been approved for the treatment of any orthoflavivirus infection [13,14]. This gap has prompted growing interest in novel therapeutic strategies, including relevant viral RNAs as targets for intervention. Although proof-of-concept studies demonstrate that RNA structures can be selectively targeted *(*e.g., for HCV IRES [15–17]), progress has been limited by the dynamic nature of RNA folding and the incomplete understanding of RNA structures and their functions [18,19]. Here, we study the conservation of a critical viral RNA structure required for orthoflavivirus RNA replication and evaluate its potential as a target for antiviral intervention.

Orthoflavivirus genomes replicate in the cytoplasm of infected cells within specialized membranous compartments induced during infection [20,21]. Viral RNA synthesis is mediated by the enzymatic activity of the nonstructural protein NS5. This protein contains two domains, the RNA-dependent RNA polymerase domain (RdRp), which catalyzes de novo synthesis of both negative- and positive-strand viral RNAs [22–26], and the methyltransferase domain (MTase) responsible for RNA methylation [27–29]. Other viral non-structural proteins also participate in the RNA replication process [30].

Two decades ago, our laboratory elucidated the mechanism of DENV RNA synthesis, which requires three essential RNA elements within the viral genome [31]. First, a promoter element known as Stem-Loop A (SLA), located at the 5′ end of the genome, binds the viral polymerase NS5 and directs initiation of RNA synthesis [31]. Second, complementary sequences located at the 5′ and 3′ ends of the genome mediate long-range RNA-RNA interactions that promote genome cyclization, a prerequisite for initiation of RNA synthesis at the 3′ end of the RNA [32–34]. Third, a highly structured 3′ stem-loop (3′SL) undergoes a conformational rearrangement upon genome cyclization, exposing the 3′ terminus to serve as the template for initiation of negative-strand RNA synthesis [35–39]. Although this mechanism was initially defined using the DENV model (Fig 1B) all three RNA structural elements are present across known orthoflavivirus genomes, strongly suggesting that the mechanism of RNA synthesis is conserved throughout the genus.

The function of the SLA for viral RNA synthesis has been experimentally validated in multiple viruses, including DENV, ZIKV, TBEV and WNV [40–42]. Several atomic-resolution structures of SLA alone and in complex with NS5 using X-ray crystallography, NMR and Cryo EM provided information about the SLA-NS5 binding mode [43–45]. The available structures of the SLA-NS5 complex identify two interaction sites: one at the top loop (TL) of the SLA that is in contact with residues of the RdRp domain and the other at the 5’ end of the RNA that interacts with the MTase domain (Fig 1C). Binding NS5 to the SLA leads to the activation of the viral RdRp for de novo initiation of RNA synthesis by a mechanism still unclear. Given its essential role in viral replication, we examine the structure and function of the SLA and its potential as an antiviral target.

In this study, we demonstrate that the SLA of DENV or ZIKV can be functionally replaced by the corresponding SLA from any other pathogenic orthoflavivirus (MBFV or TBFV). Chimeric viruses, carrying heterologous SLAs, replicate with distinct efficiencies, revealing both shared and virus-specific requirements for optimal viral RNA replication. Through detailed structure-function analyses, we identify essential SLA sub-elements that support a common RNA-NS5 binding mode and provide insights into sub-structure co-evolution that distinguish MBFV from TBFV. We also found that SLAs from ISFV are non-functional in the context of DENV or ZIKV, despite their capacity to bind NS5. Based on the shared functional features of SLAs from pathogenic orthoflaviviruses, we screened an RNA-focused small-molecule library for compounds that bind the DENV SLA. Using a fluorophore-displacement assay, we identified several compounds that exhibit significant inhibition of DENV replication at concentrations with no detectable cytotoxicity. One of these compounds was found to display inhibition of replication of different orthoflaviviruses, including ZIKV, YFV, and Langat virus (LGTV), a TBFV. Together, these results identify the orthoflavivirus SLA as a conserved RNA element and a promising target for antiviral intervention.

## Results

### The SLAs are functionally conserved among all pathogenic orthoflaviviruses

To evaluate structural and functional features of SLAs from orthoflaviviruses, we first performed sequence and structure alignments using available complete viral 5’UTRs. We built 2D and 3D models of the SLAs with sequences from mosquito-borne, DENV and YFV clades, (MBFV); tick-borne (TBFV); and insect-specific, dual host-like and classical subgroups (dhl-ISFV and c-ISFV, respectively) (Fig 1D). Low overall sequence conservation was observed with few nucleotides of SLA presenting a conservation >70% (Fig 1E). Despite this low sequence conservation, structural sequence alignments and 3D modeling of all orthoflavivirus SLAs indicate a common overall fold. The SLA structures include a top stem-loop (TSL), a bottom stem (BS), and a side loop (SL) or side stem-loop (SSL) connected by a 3-way junction (3WJ) (Fig 1F). This is consistent with the structure previously determined by RNA chemical probing and 3D structure determination by X-ray crystallography, NMR and Cryo-EM for DENV and ZIKV [43–49]. Members of the c-ISFVs lack a SSL, instead they bear a SL (Fig 1D). Structural sequence alignments indicate invariable nucleotides at defined regions of the SLA, including the 5’ end, the TL, certain positions of the 3WJ, and a U bulge within the BS (Fig 1G).

**Fig 2:**
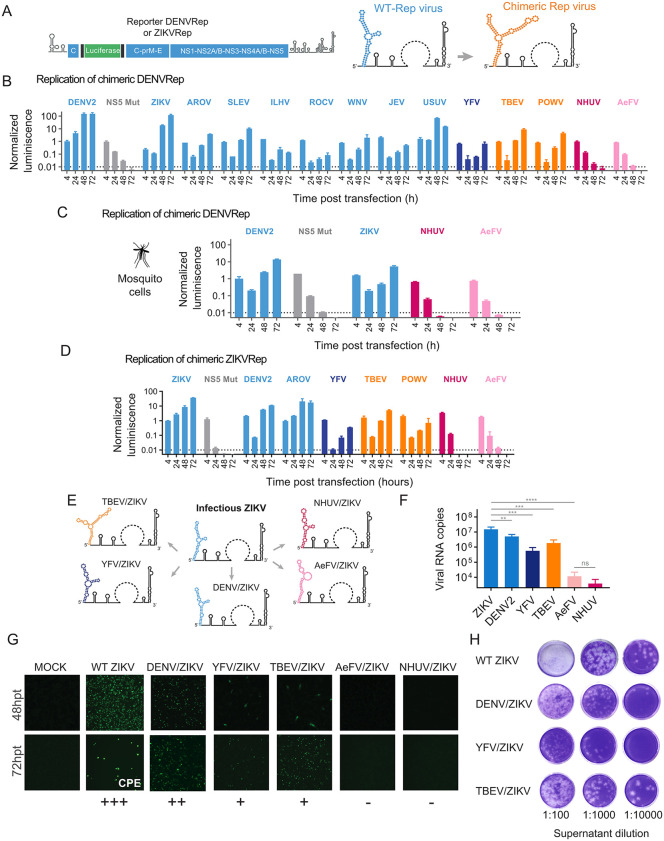
Functional conservation of SLA among pathogenic orthoflaviviruses. **A.** Schematic representation of DENV and ZIKV reporter genomes (DENVRep and ZIKVRep), on the left; and representation of chimeric viruses with replacement of the SLA structure, on the right. **B.** Replication of chimeric viruses in the context of the DENVRep. Normalized luciferase activity as a function of time is shown in BHK cells for transfected RNAs corresponding to the DENVRep WT, chimeric viruses and NS5Mut, as indicated in each case. Replication of reporter viruses was classified integrating the temporal behavior of the luciferase signal and comparisons with controls (WT and NS5 mut) as replicative or non-replicative. Statistical comparisons were performed using two-tailed Welch’s t-tests, considering three biological replicates (see Materials and Methods). **C.** Replication of chimeric viruses in the context of the DENVRep in mosquito C6/36 cells. Normalized luciferase activity is shown as a function of time. Statistical analysis was done as in **B. D.** Replication of chimeric viruses in the context of the ZIKVRep. Normalized luciferase activity as a function of time is shown in BHK cells for transfected RNAs corresponding to the ZIKVRep WT, chimeric viruses and NS5Mut, as indicated in each case. Statistical analysis was done as in **B. E.** Schematic representation of the design of chimeric viruses in the context of ZIKV infectious clone. **F.** Quantification of viral RNA copies by RT-PCR from secreted particles harvested in the supernatant of transfected cells 3 days post transfection for the viruses indicated in each case. Average and standard deviations from two independent experiments are shown. RNA copies were compared using one-way ANOVA test followed by Tukey’s multiple comparison test. **** indicates p-value<0.0001; *** indicates adjusted p-value <0.001; ** indicates adjusted p-value <0.01, ns indicates adjusted p-value >0.05. **G.** Immunofluorescence assay showing the propagation of ZIKV WT and chimeric viruses as a function of time in BHK cells. Cells were labeled with specific ZIKV anti NS3 antibodies. Underneath symbols represent the extend of viral propagation. Cytopathic effect (CPE) is indicated. **H.** Representative images of plaque assays formed by chimeric viruses. For each virus, three images are included showing results from 3 consecutive serial dilutions of the supernatant obtained at 72 hours post transfection. Mosquito image from public domain clip-art taken from NIH Bio Art. https://bioart.niaid.nih.gov/bioart/162.

Motivated by the structural conservation observed among SLAs from different orthoflaviviruses, we asked whether the entire SLA from DENV could be functionally replaced by an SLA from viruses belonging to the same or distinct ecological groups. To address this question, we used previously described replication-competent DENV and ZIKV reporter genomes encoding Renilla luciferase (DENVRep and ZIKVRep; Fig 2A) [50,51]. Transfection of in vitro-transcribed, capped viral RNAs of these constructs produce luciferase activity that, when measured over time, reflects translation of the input RNA and subsequent RNA replication. Comparison with a replication-defective control RNA carrying a mutation in the catalytic site of NS5 (NS5Mut) allows discrimination of the onset and magnitude of viral RNA amplification

To generate chimeric DENVRep constructs, the complete DENV SLA was replaced with SLAs derived from emerging and re-emerging MBFV that pose significant risks to human health, including ZIKV, JEV, Aroa virus (AROV), Saint Louis encephalitis virus (SLEV), Usutu virus (USUV), West Nile virus (WNV), Ilhéus virus (ILHV), Rocio virus (ROCV), and yellow fever virus (YFV). SLAs from human-pathogenic tick-borne orthoflaviviruses, including tick-borne encephalitis virus (TBEV) and Powassan virus (POWV), were also employed. Additionally, to account for ecological diversity, we included SLAs from Nhumirin virus (NHUV) a dhl-ISFV, and Aedes flavivirus (AeFV) a c-ISFV (Fig 2B).

Several considerations were taken into account during chimera construction. First, particular attention was paid to the sequence junction between the heterologous SLA and the DENV genome to ensure that the conserved AG dinucleotide at the 5′ end of the genome was not predicted to participate in base pairing within the bottom stem of the SLA [52]. In addition, previous studies have shown that the MTase activity (N-7 methylation) of NS5 from certain MBFV requires specific nucleotides at positions two and three of the viral genome [53]. Sequence analysis revealed variability at the third nucleotide position: whereas DENV, ZIKV, AROV, WNV, and YFV conserve a U, SLEV, JEV, USUV, ROCV, and ILHV contain an A at this position. To ensure compatibility among chimeric constructs, a U was maintained at the third nucleotide position. For more details about construction of chimeric viruses see supplementary information (S1 Fig).

Viral capped RNAs corresponding to DENVRep WT, the replication-defective NS5Mut control, and each of the chimeric constructs were generated by in vitro transcription using a modified T7 promoter to ensure the authentic GpppAGU 5’ end viral sequence, quantified, and transfected into BHK cells. Luciferase activity was measured at 4, 24, 48, and 72 hpt. All chimeras containing SLAs derived from pathogenic orthoflaviviruses, including YFV and the TBFVs, displayed clear evidence of viral RNA replication when compared with the NS5Mut control (Fig 2B). The normalized luciferase activity at 72 hpt was significantly higher than that of the NS5Mut control. Several chimeric viruses exhibited a replicative but suboptimal phenotype, such as the chimeras containing SLAs from ILHV, ROCV, JEV, and YFV (Fig 2B). For statistical analysis, see Material and Methods. In contrast, chimeras carrying SLAs from the dual-host-like and classic ISFV (NHUV and AeFV, respectively) failed to replicate, exhibiting luciferase levels not significantly different to those of the NS5Mut control (Fig 2B).

Because ISFV replicate exclusively in insect cells, we examined whether the lack of replication observed for NHUV and AeFV chimeras was due to host cell restriction. To this end, capped viral RNAs from DENVRep WT, NS5Mut, the ZIKV SLA chimera, and the ISFV chimeras (NHUV and AeFV) were transfected into mosquito C6/36 cells. Luciferase measurements revealed efficient replication of the ZIKV SLA chimera, whereas both ISFVs remained replication incompetent (Fig 2C). These results indicate that the inability of ISFV SLAs to support replication in the DENV context is not due to host cell specificity.

Our data suggest that the SLA from any pathogenic orthoflavivirus can support viral RNA replication in the context of DENV, consistent with functional conservation of this RNA structure. To further validate this conclusion, we generated analogous chimeras in the context of ZIKVRep. SLAs from representative orthoflaviviruses were used to replace the native ZIKV SLA, including SLAs from DENV, AROV, YFV, TBEV, POWV, NHUV, and AeFV. Consistent with the results obtained in the DENV context, chimeras with SLAs from MBFV and TBFV supported RNA amplification in the context of ZIKV, whereas chimeras with SLAs from ISFV were inactive (Fig 2D).

To further confirm our findings obtained with DENV and ZIKV reporter systems, we analyzed SLA function in the context of infectious viruses. Chimeric viruses were generated using an infectious ZIKV clone previously constructed in our laboratory [50], in which the native ZIKV SLA was replaced with the SLA from DENV, YFV, TBEV, NHUV, or AeFV (Fig 2E). Capped viral RNAs were transfected into cells, and viral replication and propagation were assessed by RT-PCR quantification of RNA in secreted viral particles, by immunofluorescence assays (IFA) detecting the viral protein NS3, and by plaque assays (Fig 2F, 2G and 2H). Viruses containing SLAs from DENV, YFV, or TBEV were able to replicate and propagate, although with reduced efficiency compared with WT ZIKV (Fig 2F). In contrast, chimeras carrying SLAs from ISFVs failed to replicate, showing no detectable viral proteins (Fig 2G).

Together, these results demonstrate that SLAs derived from pathogenic MBFV or TBFV are functionally conserved and support viral RNA replication across heterologous genomic contexts. Despite sequence divergence, these SLAs retain shared structural and functional features that enable productive interaction with the viral replication machinery of DENV and ZIKV, consistent with a conserved mechanism of SLA recognition by NS5 during cap methylation and viral RNA synthesis.

### Structure-function relationships among orthoflavivirus SLAs

We found that SLAs from MBFVs and TBFVs support viral RNA replication in the context of DENV and ZIKV, albeit with different replication efficiencies, whereas the ones from ISFV are not compatible with DENV or ZIKV. To better understand structure-function relationships of sub-elements of SLAs, we analyzed group-specific and virus-specific features in the 2D and 3D models of SLAs from different orthoflaviviruses.

**Fig 3:**
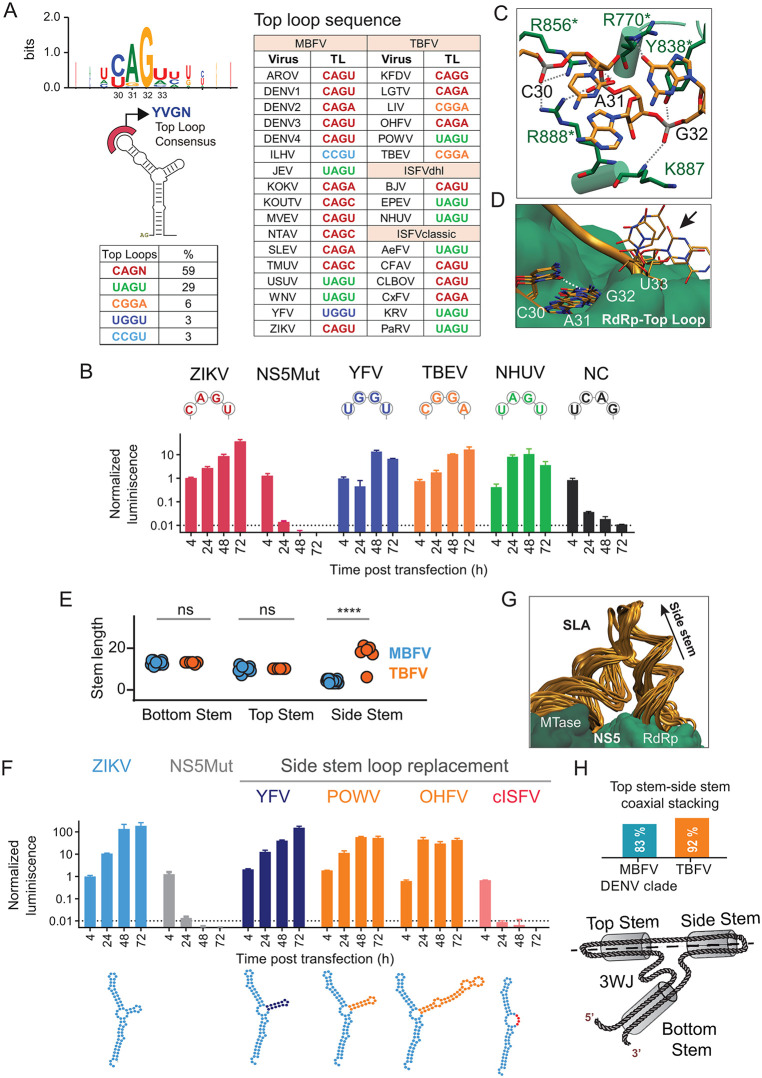
Structure-function relationships of the TL and SS in orthoflaviviruses. **A.** Sequence conservation of MBFV, TBFV and ISFV TLs. Logo obtained from alignment showing the frequency of individual nucleotides. On the right, table showing the TL nucleotide sequence for each virus. Members of the MBFV, TBFV and ISFV (classic and dual host like) groups are indicated. **B.** Impact of TL sequences on ZIKVRep RNA replication. Normalized luciferase activity as a function of time is shown for the WT, mutant ZIKVRep and the NS5Mut in BHK cells. The TL sequence for each mutant is indicated on the top. The color code corresponds to each TL sequence as indicated in the table above. Replication of reporter viruses was classified integrating the temporal behavior of the luciferase signal and comparisons with controls (WT and NS5 mut) as replicative or non- replicative. Statistical comparisons were performed using two-tailed Welch’s t-tests, considering three biological replicates (see Materials and Methods). **C.** Snapshot from the MD simulation showing contacts between TL nucleotides and residues of the RdRp thumb domain of NS5 conserved during 1-microsecond MD trajectories (3 replicas). SLA TL nucleotides C30, A31, G32 are indicated. The thumb region is shown. Residues with 100% conservation among orthoflaviviruses are marked with an asterisk. **D.** Snapshots from MD simulations superimposed to show conformations of SLA TL nucleobases (C30, A31, G32 and U33). The interaction between the first and third bases (dashed line) is maintained during all three microsecond simulations. **E.** Length of the TS, BT and SS grouped according to ecological group. Lengths were compared using the Mann–Whitney unpaired test. **** indicates adjusted p-value <0.0001; ns indicates adjusted p-value >0.05. **F.** Impact of different SSLs on viral replication. Normalized luciferase activity as a function of time for the ZIKVRep WT, chimeras and the NS5Mut in BHK cells as indicated in each case. Underneath representation of SLAs with the corresponding SSL in different colors. Statistical analysis was done as in **B. G.** Superimposed snapshots of the MD simulation highlighting the position of the SS and TS-SS stacking observed in 97% of configurations. **H.** Fraction of SLA sequences with at least 1 3D model with coaxial TS-SS stacking in each ecological group. At the bottom a cartoon representation of the 3WJ and the coaxial TS-SS stacking.

We first examine the properties of the TL. The nucleotide sequence at the TL has been shown to be critical for DENV SLA-dependent NS5 polymerase activity, demonstrated by in vitro de novo RNA synthesis and viral infections [31,46]. Functional studies also assigned an essential role of TL sequences in WNV [54]. Structural analyses of the DENV SLA in complex with NS5 identified direct contact between the thumb domain of the RdRP and specific nucleotides of the TL (Fig 1C) [43,45,55]. Thus, we examine in more detail the TL sequence conservation among SLAs from orthoflaviviruses representing the different ecological groups. A consensus sequence YVGN was observed, in which *Y* represents a pyrimidine (C/U), *V* any nucleotide except U, and *N* any nucleotide (Fig 3A). The TLs were restricted to eight unique sequences in natural isolates (only 3% of the possible sequence space): CAGU, CAGA, CAGG, CAGC, UAGU, CGGA, UGGU, CCGU (Fig 3A). CAGN was the most prevalent motif (59% of the sequences), followed by UAGU (Fig 3A). Interestingly, the TL sequence variation did not correlate with ecological group classification. For example, UAGU was found in a MBFV (WNV), a TBFV (POWV) and an ISFV (NHUV) (Fig 3A).

To investigate the functional requirement of TL sequences, we used the ZIKVRep. We engineered chimeric viral RNAs in which the WT TL sequence was replaced with that of representative viruses: DENV, YFV, TBEV, NHUV or with a non-consensus UCAG sequence (NC) lacking the conserved G at the third position, which is not observed in nature (Fig 3B). The TL sequence of AeFV is the same as the one of NHUV (UAGU) (Fig 3A). Quantified viral RNAs from WT ZIKVRep and chimeric viruses were transfected into BHK cells alongside the control NS5Mut. RNAs with SLAs harboring TL found in natural isolates supported efficient viral RNA replication, as indicated by increasing luciferase activity over time. In contrast, the viral RNA containing the NC TL failed to replicate, displaying luciferase kinetics comparable to that of NS5Mut control (Fig 3B). These findings indicate that the TL of the SLA tolerates limited sequence variability, with only a restricted set of naturally occurring motifs supporting viral RNA replication, which is independent of viral ecological groups.

The invariable G found at the third position in the TL of all orthoflaviviruses coincides with specific interactions between this nucleobase with R770 and K841 in the RdRp thumb region [43,45]. To explore the stability of these specific interactions, we performed molecular dynamics (MD) of the NS5-SLA complex. The specific nucleobase-side chain interaction of the third TL nucleotide (G32) with R770 was found to be stable throughout all three microsecond simulations, with a distance between the carbonyl oxygen of the guanine base and the central carbon of the guanidinium group of the side chain of 3.6 +/- 0.6 Å (Figs 3C and S2). In contrast, the interaction between the nucleobase and K841 was not conserved in any of the three simulations (average nucleobase-sidechain distance of 7 +/- 1 Å) (S2 Fig). Other residues within the thumb domain of the RdRp that establish stable interactions with the RNA backbone of the first three nucleotides of the TL are highlighted in Fig 3C. From the NS5 perspective, residue conservation among all orthoflaviviruses was observed. The amino acid side chains of the RdRp that maintain stable interactions with the TL (R856, R770, Y838, and R888, Fig 3C) are 100% conserved among orthoflaviviruses. The partial sequence and functional conservation of the first and second nucleotides of the TL might be explained by their involvement in protein-RNA interactions mediated through the RNA backbone rather than the nucleobases. The conformation of the RNA backbone is stabilized by an interaction between the first and third nucleobases that persists during all three microsecond MD trajectories (Fig 3D). This is consistent with the restricted sequence variability of the first position to either C or U; both nucleobases have a carbonyl group at the C2 position of their pyrimidine ring that engages in this hydrogen bond with the conserved G in the third position. On the other hand, the fourth nucleotide adopts multiple conformations facing away from the protein surface (Fig 3D), consistent with the natural large variability observed at this position. Together, the replication profiles of ZIKVRep genomes carrying different natural TL sequences, along with structural and sequence analyses, support a conserved mode of interaction between the TL and the thumb domain of the RdRP across orthoflaviviruses.

Then, we investigated structures and functions of the stems that form the SLA. Within the BS, a U bulge was originally identified in DENV as essential for RNA replication. Substitution U/A in this position resulted in revertant viruses that restored the sequence [46]. We observed an absolute conservation of the U bulge in different viruses, supporting an important function (Fig 1D). A major difference among orthoflavivirus SLAs is the presence and length of the side stem-loop (SSL) (Fig 1D). Predicted 2D models from pathogenic virus SLAs display a SSL, whereas the SLAs from c-ISFV display a side loop (SL), representing an important difference among distinct viral ecological groups. The side stem (SS) could be in a dynamic equilibrium with a SL based on previous observations, including intermediate reactivity in SHAPE assays [43,45–49]. In MBFVs, the length of the SS ranges from 3 to 5 base pairs, whereas in TBFVs, it can extend up to 21 base pairs (Fig 3E). To assess the functional relevance of the presence and length of a SS, we generated chimeric ZIKVRep RNAs by replacing the SSL of ZIKV with that of two TBFVs, POW or OHFV, as well as YFV, and a c-ISFV SL sequence (Fig 3F). Viral RNAs were quantified and transfected into BHK cells together with the non-replicative control. Luciferase activity measured as a function of time indicated that constructs with SLA containing any SSL supported viral RNA amplification like the WT virus. In contrast, the viral RNA with the SLA carrying the c-ISFV SL sequence did not show signs of RNA replication (Fig 3F). Structural analysis of the SLA in complex with NS5 indicates that the SSL protrudes away from the protein surface providing an explanation for why the length of this element does not impact on the SLA function on promoting viral RNA amplification (Fig 3G). However, the presence of this element appears to be critical.

A co-axial stacking of the SS with the TS observed in the DENV SLA-NS5 structure was found to be stable throughout all of the three independent microsecond MD simulations (97% of conformations). Interestingly, this co-axial stacking of the TS and SS was also observed in the 3D models of SLAs from MBFV and TBFV, with the exception of YFV (Fig 3H). These results suggest that the SS-TS stacking is a common feature that may be important for SLA function.

**Fig 4:**
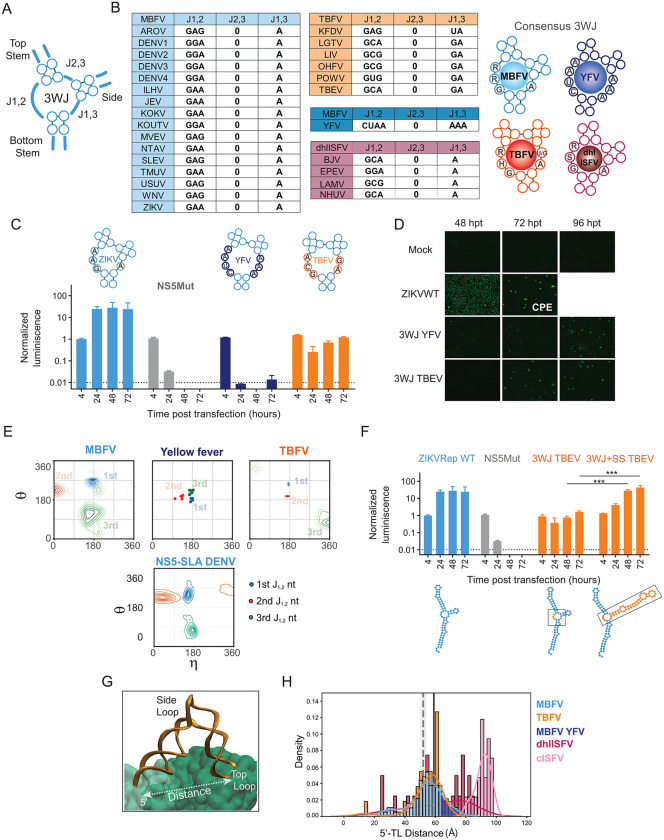
Structure-function analysis of the three-way junction of orthoflavivirus SLAs. **A.** Schematic representation of the 3WJ, indicating the three helices and the single-stranded linkers that connect them: J_1,2_; J_2,3_ and J_1,3_. **B.** Sequences of 3WJ in each member of the different groups as indicated, MBFV, TBFV and dhl-ISFV. On the right, the 3WJ consensus sequence and a representation of the structure for each group, as indicated. **C.** Normalized luciferase activity as a function of time for ZIKVRep WT, 3WJ chimeras and the NS5mut in BHK cells as indicated. Replication of reporter viruses was classified integrating the temporal behavior of the luciferase signal and comparisons with controls (WT and NS5 mut) samples were classified as replicative or non- replicative. Statistical comparisons were performed using two-tailed Welch’s t-tests, considering three biological replicates (see Materials and Methods). **D.** Immunofluorescence assay showing the propagation as a function of time of ZIKV WT and the viruses carrying the 3WJ of YFV and TBEV as indicated, BHK cells were labeled with ZIKV anti NS3 antibodies. Cytopathic effect (CPE) is indicated. **E**. η and θ pseudo torsion angles corresponding to the 3WJ nucleotides of J_1,2_ of the SLA 3D models and of the DENV2 during the MD simulation. **F.** Normalized luciferase activity as a function of time for ZIKVRep WT, NS5Mut and chimeric viruses carrying the 3WJ or 3WJ + SS of TBEV as indicated. Comparison between de 3WJ TBEV and the 3WJ + SS TBEV at 48 and 72 hpt was performed using two-tailed Welch’s t-tests. *** indicates adjusted p-value <0.001. **G.** Representation indicating the distance between the TP and 5’ end of the RNA in the RNP complex. **H.** Distribution of 5’-TL distance (in Å) in 3D models of SLAs from MBFV (blue), TBFV (orange), dh-ISFV (dark pink) and c-ISFV (light pink) ecological groups. Each histogram was calculated using the seaborn hist plot function with 40 bins and normalized such that the total area is 1. A kernel density plot is included for ease visualization of the distributions (bandwidth determined using Scott’s rule) [58]. The region of the kernel density plot of the MBFV group shaded in dark blue highlights the values for YFV (63-73 Å). Vertical lines reference corresponding 5’-TL distances from the NS5-SLA structure (PDB 8gzp, 59 Å, solid black line) [45], and from the MD simulations of the complex (52 ± 2 Å, vertical dashed line and surrounding shaded region).

The three stems converge in the 3WJ, which is important in defining the 3D architectural arrangement of the SLA. In the 3WJ, short single-stranded linkers connect the three helices (junctions J_1,2_, J_2,3_ and J_1,3_, Fig 4A) and the lengths of the linkers strongly influence junction geometry, coaxial stacking, and stem flexibility. The 3WJs can be classified as Types A, B, or C based on which helices stack coaxially and on lengths of these junctions [56]. DENV2 SLA 3WJ falls into Type C, in which the TS and SS are stacked, the J_1,2_ bottom-top linker is longer than the J_1,3_ side-bottom linker, and the top-side linker J_2,3_ is absent (0 nt). Conserved secondary structure-sequence alignment across MBFV and TBFV SLAs revealed group-specific 3WJ motifs with YFV displaying a unique pattern (Fig 4B). MBFVs SLAs show that J_1,2_ is GAG, GAA or GGA, J_2,3_ is absent, and J_1,3_ is a single conserved A. In the case of YFV, J_1,2_ is CUAA, J_2,3_ is absent, and J_1,3_ is AAA. In TBFVs, J_1,2_ follows a GHR consensus sequence (where H is anything except G, and R a purine), J_2,3_ is absent, and J_1,3_ contains two nucleotides, GA or UA. The 3WJ of dhl-ISFV displays a pattern similar to that of MBFV, but the J_1,2_ is GCA, GCG or GGA (Fig 4B).

To assess the role of the 3WJ within the SLA structure in viral replication, we generated new chimeric ZIKVRep by replacing only the 3WJ sequence of the ZIKV SLA with that of YFV (3WJ-YFV ZIKVrep) or TBEV (3WJ-TBEV ZIKVrep). Secondary structure predictions of these chimeric SLAs suggested that the overall folding of the SLA was preserved. RNA from WT ZIKVRep, the two chimeras, and NS5Mut ZIKVrep, were quantified and transfected into BHK cells. Luciferase expression levels showed that virus carrying the 3WJ-TBEV retained partial replication capacity, showing a ~ 2-log reduction relative to WT ZIKVRep at 24 hours, while the RNA with the 3WJ-YFV showed minimal replication (Fig 4C). The results suggest that the 3WJ sequence and structure is a sensitive element for SLA function. To confirm this observation, we used the same 3WJ mutations but in the context of the ZIKV infectious clone and assessed whether the chimeric viruses (3WJ-YFV ZIKV and 3WJ-TBEV ZIKV) were able to replicate and propagate in cultured cells. We followed viral propagation by IFA against the viral NS3 protein. While the ZIKV WT showed a complete monolayer infected at 48 hpt and at 96 hpt most of the monolayer showed cytopathic effect (CPE), signs of propagation of both chimeras were observed at 96 hpt, suggesting inefficient replication of these viruses, in agreement with the data obtained with the ZIKVRep (Fig 4D).

To structurally characterize the 3WJ, we analyzed the η (eta) and θ (theta) pseudo torsion angles in the 3D models of the SLAs corresponding to the nucleotides of J_1,2_. The η and θ angles represent the backbone geometry in two dimensions and permit the identification of structural motifs [57]. We observed that J_1,2_ nucleotides generally fall in the same regions of η-θ space within each ecological group, indicating a similar conformation of the 3WJs (Fig 4E). For the MBFV group, this pattern places the first, second and third nucleotides of J_1,2_ in the helical, stacked-turn, and base-twist regions, respectively. This conformation coincides with that of the DENV4 SLA in the NS5-bound structure [45], and each of the nucleotides remained in its corresponding region during all three microsecond MD simulations. The dh-ISFV 3WJ also coincides in these regions of the η-θ plot, whereas YFV clustered separately. MBFV and TBFV occupy distinct regions in η–θ space, supporting the idea that 3WJ architecture diverged among these ecological groups (Fig 4E).

We observed that introduction of the TBEV SLA into the ZIKV context delays viral replication significantly less than replacing only 3WJ nucleotides (compare Figs 2D and 4C). Several explanations could account for this difference. One possibility is that additional structural elements within the TBEV SLA compensate for the impact of the heterologous 3WJ in the chimeric virus. Given that the SSL differs markedly between MBFVs and TBFVs, we hypothesized that these two SLA sub-elements (the SSL and the 3WJ) may have co-evolved within distinct ecological groups.

To test this hypothesis, we generated a new chimeric ZIKVRep in which both the 3WJ and the SSL of ZIKV were replaced by the corresponding elements from TBEV. Capped viral RNAs were generated and transfected into cells, and replication was monitored by luciferase activity. The chimera containing both TBEV elements replicated significantly more efficiently, about two orders of magnitude, higher than the virus carrying only the TBEV 3WJ (Fig 4F). These results indicate that although 3WJ sequences of SLAs have diverged among viruses from different ecological groups, co-evolution with other SLA sub-elements may preserve promoter function.

The available NS5–SLA structure indicates that the MTase domain contacts the 5′ end (BS), whereas the RdRp engages the TL. Thus, the relative orientation of the TSL and BS, and the distance between the 5′ end and the TL, are likely critical determinants of NS5 binding (Fig 4G). In the cryo-EM structure of the DENV NS5–SLA complex, this distance is approximately 59 Å and converges to an average value of 52 + /- 2 Å during MD simulations. The distributions of this distance in 3D models of free SLAs from pathogenic orthoflaviviruses (MBFV and TBFV) are centered close to these values, with average values of 53 and 54 Å for the MBFV and TBFV groups, respectively, except for YFV with predicted distance centered at 65 Å (Fig 4H). In contrast, SLAs from the c-ISFV group display a markedly distinct distribution centered at ~91 Å, with no overlap with the pathogenic groups (Fig 4H). Together, these results indicate that the 5’-TL distance of SLAs is another common structural feature among pathogenic orthoflaviviruses.

Together, our functional and structural analyses indicate that productive viral replication requires not only conserved structures and specific sequences within individual SLA sub-elements, but also their coordinated organization. Although certain SLA features have diverged in a group-specific manner, the combination of these elements maintains a conserved global architecture of the SLA that enables heterologous NS5 recognition and viral RNA replication.

### The binding of ISFV SLAs to NS5 is not sufficient to support RNA replication

Members of the ISFV group are divided into two phylogenetically distinct subgroups. While the dual host-like subgroup is genetically closer to MBFVs, the classical subgroup is more distantly related, a divergence that is reflected in the structure of their SLAs (Fig 1A and 1D). Comparative analyses revealed distinct structural and functional features among SLA sub-elements in these viruses, which may account for the lack of SLA functionality observed in the context of DENV and ZIKV.

**Fig 5:**
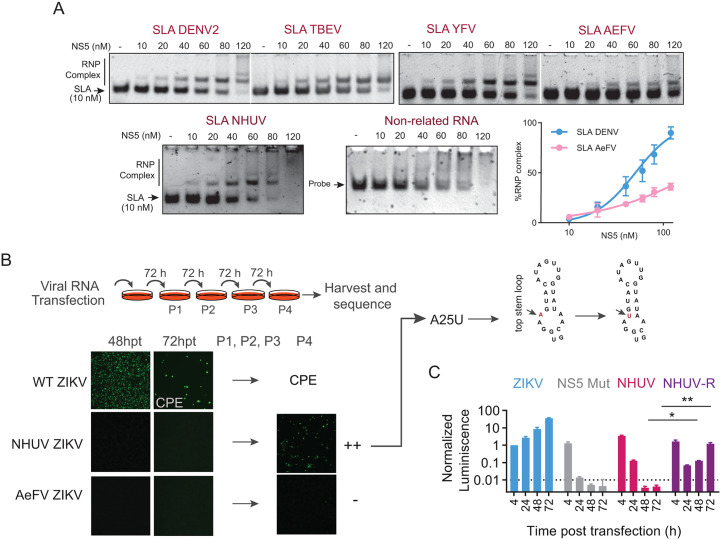
NS5 binding properties of different SLAs and in culture evolution of ZIKV carrying ISFV SLAs. **A.** SLA-NS5 interaction by EMSA. Representative gels showing binding properties of purified NS5 protein to SLA from DENV, TBEV, YFV, AeFV, NHUV and an unrelated RNA as indicated. Mobility of the free SLA and the RNP complex is shown. The NS5 concentration is indicated on the top, and the SLA concentration is 10 nM. A plot with binding curves of NS5 to the indicated SLAs based on the bound RNA fraction from the EMSA is shown at the bottom right. A sigmoidal four-parameter logistic curve model was fitted to data. **B.** Search for adaptive mutations of non-replicating chimeric viruses carrying ISFV SLAs. On top, schematic representation of experimental procedure showing serial passages (P1, P2, P3 and P4). At the bottom, immunofluorescence assay images of transfected mammalian cells with ZIKV infectious RNA and the chimeric viruses (NHUV ZIKV and AeFV ZIKV) as a function of time. NHUV ZIKV replicates after serial passages. Sequence of rescued virus reveals a mutation at the top stem, A26U. On the right, secondary structure of the NHUV SLA indicates the location of the A26U mutation. **C.** The mutation obtained is sufficient to support viral RNA replication (NHUV-R ZIKV). Normalized luciferase levels as a function of time for the ZIKVRep WT, NHUV-ZIKVRep, NHUV-R ZIKVRep chimeras and NS5Mut are shown. Comparison between de NHUV and NHUV-R chimeric virus at 48 and 72 hpt were performed using two-tailed Welch’s t-tests. ** indicates adjusted p-value <0.01; * indicates adjusted p-value <0.05.

To further investigate the basis for the absence of replication observed in chimeric MBFVs carrying ISFV-derived SLAs, we examined the ability of these SLAs to form heterologous complexes with DENV NS5. Protein-RNA interactions were analyzed using electrophoretic mobility shift assays (EMSA). Recombinant purified DENV NS5 was produced, and RNA molecules corresponding to the SLAs from DENV, YFV, TBEV, NHUV, and AeFV were generated. SLA RNAs were used at a fixed concentration (10 nM), while NS5 was titrated from 10 to 120 nM (Fig 5A).

As a control, EMSA performed with the DENV SLA revealed the formation of a defined ribonucleoprotein (RNP) complex, consistent with previous reports [31,59]. Across most NS5 concentrations, a single discrete complex was observed, whereas at higher NS5 concentrations slower-migrating complexes appeared, consistent with the binding of multiple NS5 molecules [43,54,59]. Comparable RNP complexes were detected with SLAs from DENV, TBEV, YFV, and NHUV. In contrast, although the AeFV SLA was also capable of forming a complex with DENV NS5, its apparent binding affinity was markedly reduced (Fig 5A). A non-related RNA of identical length was included as a control for non-specific interactions; in this case, NS5 binding was detected, as expected given its RNA binding nature, but no defined RNP complex was formed.

Together, these results suggest that the NHUV SLA binds DENV NS5 with an apparent affinity comparable to that of the DENV SLA, whereas the AeFV SLA exhibits substantially weaker binding. These data suggest that a high-affinity interaction between NHUV SLA and NS5 is not sufficient to support RNA replication in the context of chimeric viruses.

To further investigate the inability of ISFV SLAs to function as promoters for MBFV RNA replication, we searched for adaptive mutations arising during serial passage in cell culture. Cells were transfected with ZIKV infectious RNAs carrying the NHUV or AeFV SLAs, along with WT ZIKV as a reference, and subjected to successive passages every 72 hours for a total of 15 days. Viral replication was monitored by IFA (Fig 5B). The chimera carrying the NHUV SLA displayed signs of viral propagation by passage 4 (12 days), whereas no propagation was detected for the virus carrying the AeFV SLA, even when experiments were extended to 20 days.

To determine whether replication of the NHUV SLA chimera was associated with adaptive mutations, viral RNA was extracted from supernatants collected at passage 4, amplified by RT-PCR, and the 5′UTR and NS5 coding regions were sequenced. Independent experiments identified a single nucleotide substitution within the NHUV SLA. This mutation mapped to the TS of the SLA structure, which could form an additional base pair (Fig 5B). To directly test whether this mutation restored SLA function in the context of ZIKV, the identified change was introduced into the non-replicative ZIKVRep-NHUV construct (Fig 5C). Capped RNAs corresponding to WT ZIKVRep, the original NHUV SLA chimera, and the rescued NHUV SLA (NHUV-R) were transfected into cells, and RNA replication was monitored by luciferase activity. The data show that the NHUV-R construct supported viral RNA replication, showing luciferase levels near 3 logs higher than the parental chimera at 72 h (Fig 5C).

Together, these results demonstrate that the NHUV SLA structure can acquire functional compatibility with the MBFV replication complex through minimal structural adaptation, whereas the AeFV SLA lacks essential features that were not rescued through cell culture-driven evolution.

### Small molecules binders of SLA as inhibitors of viral replication

**Fig 6:**
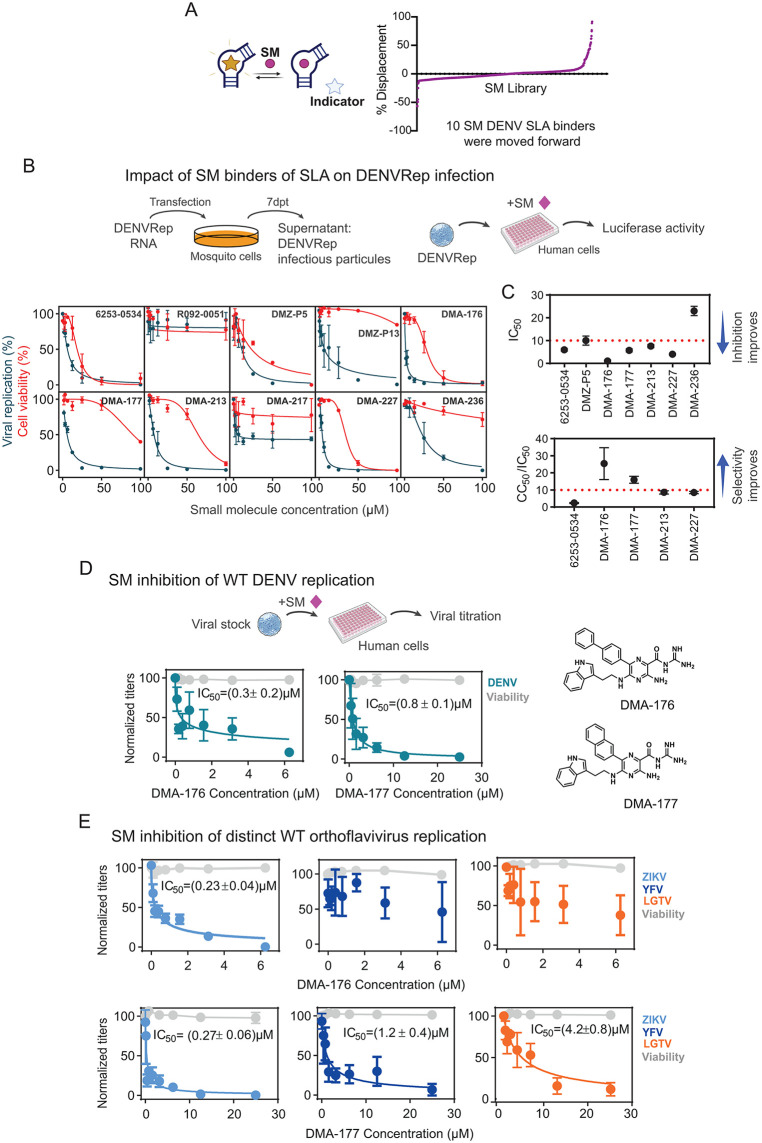
Small molecules inhibition of orthoflavivirus infection. **A.** Left: Representation of the indicator displacement assay using the TOPRO-1 dye. When the indicator (star) is bound to RNA, signal is enhanced. Displacement of the indicator by a competitive small molecule (sphere) diminishes signal. The assay was used to screen the RNA-targeted library against DENV2 SLA. Right: Waterfall plot of screening results at 10 µM small molecule. **B.** Selected SMs that bind DENV2 SLA were use to evaluate impact on DENVRep replication. At the top, schematic representation of DENVRep viral particle production and screen of viral RNA replication measuring luciferase activity. At the bottom, viral inhibition and cell viability curves for each molecule are shown as indicated. **C.** Viral inhibition and selectivity of the selected SMs. Plots representing apparent IC_50_ and CC_50_/IC_50_ are shown. **D.** Inhibition of DENV2 WT by DMA-176 and DMA-177. At the top, schematic representation of the assay and, at the bottom, plots showing viral titer reduction (green) and cell viability (grey) as a function of SM concentration. Curves are mean of three independent experiments. Right: Structures of DMA-176 and DMA-177. **E.** Effect of DMA-176 and DMA-177 on the infection of ZIKV WT, YFV 17D and LGTV. Viral titers were measured as a function of SM concentration. Cell viability (grey) is also shown. Sigmoidal four-parameter logistic curve models were fitted to luciferase, viral titers and cell viability data to determined apparent IC_50_ and CC_50_. Drawings used in this figure include public domain clip-art images taken from NIH Bio Art: https://bioart.niaid.nih.gov/bioart/7 and https://bioart.niaid.nih.gov/bioart/156.

To identify small molecule binders of SLA, we screened an RNA targeted library of commercial small molecules (DRTL) and synthetic small molecules reported in recent work [60–62]. An indicator displacement assay (IDA) using the TOPRO-1 dye was determined to be suitable for screening based on a Z’-score of 0.54 (Z’-score > 0.4 is acceptable) (Fig 6A). Plates containing the TOPRO-1 dye and DENV2 SLA were treated with 10 µM small molecule and the percent displacement of the dye calculated. Data from three independent screens were averaged, and hits were identified as small molecules with percent fluorescent indicator displacement (%FID) values greater than 20% (46 hits, 4.7% hit rate). Of these hits, 10 binders were selected for further testing based on %FID and hit frequency in other assays.

Selected binders were further evaluated for antiviral activity. Initial screening was performed using the DENVRep system, which produces infectious particles encoding luciferase. Viral stocks were generated in mosquito cells and used to infect human cells in the presence of increasing concentrations of SMs (Fig 6B). Viral replication was quantified by luciferase activity, and cytotoxicity was assessed in parallel for each compound. Apparent half-maximal inhibitory concentrations (aIC₅₀) and apparent cytotoxic concentrations (aCC₅₀) were determined (Fig 6C). Molecules displaying the highest selectivity indices were selected for further analysis (dimethyl amiloride DMA-176 and DMA-177) using WT DENV.

Human A549 cells were infected with DENV at a multiplicity of infection (MOI) of 0.1 in the presence of increasing concentrations of DMA-176 or DMA-177. Viral particle production in culture supernatants was quantified by plaque assay, and compound cytotoxicity was evaluated using CellTiter-Glo. Both compounds inhibited DENV replication at submicromolar concentrations, with aIC₅₀ values of 0.3 ± 0.2 μM for DMA-176 and 0.8 ± 0.1 μM for DMA-177.

We next examined whether these compounds inhibited replication of other orthoflaviviruses. Infectious stocks of ZIKV, YFV, and Langat virus (LGTV; a tick-borne orthoflavivirus) [63] were generated, and human cells were infected at an MOI of 0.1 in the presence of increasing concentrations of DMA-177 or DMA-176 (Fig 6D and 6E). DMA-176 inhibited ZIKV replication but showed no significant inhibition of YFV or LGTV. In contrast, DMA-177 inhibited replication of ZIKV, YFV, and LGTV, indicating broader antiviral activity across orthoflaviviruses. The different effect observed with DMA-176 and DMA-177 suggest that subtle structural differences among SLAs influence compound sensitivity. Together, the results show that small molecules targeting the SLA can inhibit orthoflavivirus replication, representing a proof of principle for broad antiviral activity.

## Discussion

Here, we present evidence supporting the functional conservation of the RNA promoter SLA among pathogenic orthoflavivirus genomes. The ability of the SLA from different mosquito- and tick-borne orthoflaviviruses to support RNA replication of DENV and ZIKV, together with conserved structural and sequence features, establishes a common functional mode of promoter NS5 recognition by all these human viral pathogens. Using recombinant viruses, together with structural modeling and MD simulations, we define shared and virus-specific structure-function relationships of SLA sub-elements across orthoflaviviruses. Our data support that divergence of SLA structural elements in viruses from different ecological groups co-evolved to support a common mechanism for viral replication. Based on this universality of SLAs, we screened an RNA-focused library of small molecules for DENV SLA binders and found molecules that display inhibition not only of DENV but also of other pathogenic orthoflaviviruses.

Targeting viral RNA structures represents an emerging and promising antiviral strategy, but its success depends on a detailed understanding of RNA function and structure [64]. Here we found two DMA molecules as binders of SLA that inhibit replication of different orthoflaviviruses. DMAs have been previously reported to bind specific RNA structures of different viruses including HIV, enterovirus A71 and SARS CoV2 [65–68] showing potential as antiviral drugs. It has been shown that DMA-135 binds the SLII of the enterovirus-A71 IRES [67]. Structural and biophysical studies supported an allosteric mechanism in which DMA-135 induces a conformational change in the SLII RNA that stabilizes a ternary complex with the AUF1 protein [68]*.* We found DMA-176 and DMA-177 as binders of DENV SLA. DMA-177 showed inhibition of members of the MBFV and TBFV, providing a proof of concept for a broad range effect on orthoflvivirus infection. In contrast, DMA-176 inhibited DENV and ZIKV, without significant effect on YFV or LGTV replication. The differential impact of DMA-176 and DMA-177 could be explained by distinct structural properties of SLAs from YFV and TBFVs compared with those from other MBFVs (Figs 3 and 4). Nevertheless, more biochemical and structural studies will be necessary to define the mechanism of action of these DMAs on orthoflavivirus replication.

Consistent with our findings, previous studies have reported that SLAs of certain orthoflavivirus can be exchangeable [40,46,54,69]. However, a lack of SLA compatibility between JEV and WNV, two members of the MBFV group, has been described [54]. Although initially puzzling, this observation can be explained by the strict requirement of the WNV NS5 methyltransferase (MTase) domain for specific nucleotides at positions two and three of the viral genome (5′-AGU), whereas JEV contains a 5′-AGA motif at these positions [53]. In agreement with this interpretation, here we found that SLAs from MBFVs whose genomes naturally carry a 5′-AGA motif, including SLEV, JEV, ILHV, USUV, and ROCV, are not functional in the context of DENV, which, like WNV, contains a 5′-AGU sequence (S1 Fig). Importantly, substitution of the third-position adenine with uridine in these SLAs was sufficient to restore viral replication in all MBFV chimeric viruses (S1 Fig). Intriguingly, the SLAs of TBFVs, which all initiate with 5’AGA motif are functional in the context of DENV and ZIKV chimeras. In line with this observation, it has been previously reported that WNV NS5 is competent for in vitro N-7 methylation of POWV (a TBFV) RNA [53]. indicating that the 5′-AGA sequence can be accommodated when presented within a TBFV SLA structural context. Together, the previous observations and the one presented here support a functional compatibility of the complete TBFV SLA in the MBFV context.

In contrast to the functional compatibility observed between MBFVs and TBFVs, the SLA from the c-ISFV AeFV failed to support DENV or ZIKV replication in either vertebrate or insect cells and showed reduced binding to DENV NS5, indicating divergence of the NS5-SLA interface beyond functional compatibility. The SLA from the dhl-ISFV NHUV, which is phylogenetically closer to MBFVs, retained the ability to bind DENV NS5 with an apparent affinity comparable to that of the homologous DENV SLA (Fig 5A). The phylogenetic proximity, reflected in similar structural features, likely explains why functional compatibility could be achieved after limited adaptation in cell culture. In this regard, replication of the NHUV SLA chimeric virus was associated with selection of a single mutation in the TS. Although the molecular mechanism by which this mutation enables productive replication remains unclear, the tolerance of bulges and mismatches in the TS of orthoflaviviruses suggests that local stem stability and geometry, rather than primary sequence, may be relevant for promoter competence.

Functional, structural and computational analyses reveal divergence of SLA sub-elements from MB and TBFV SLAs. Differences in 3WJ sequence and junction lengths, as well as in the length and composition of the SSL, distinguish SLAs from these two groups of viruses. Within the same viral group, 3WJs are conserved (Fig 4A), suggesting possible interactions with host-specific factors. Previous studies have proposed that the 3WJ plays a key role in shaping the 3D conformation of the SLA [43,44]. NMR analyses indicate that the 3WJ is partially flexible, and additional structural studies have proposed that this flexibility allows the SLA to adopt distinct global architectures. Thus, the 3WJ may provide SLA movement plasticity for transitioning between different stages in the viral RNA replication process.

Despite divergence among SLA sub-elements, several key features remain conserved across MBFVs and TBFVs, including conserved coaxial stacking of the SS with the TS and restricted sequence variation within the TL (Fig 3). Although the four-nucleotide TL could theoretically generate 256 sequence combinations, only eight are observed in nature, reflecting strong functional constraints. Specific contacts between TL nucleotides and conserved RdRp residues, together with a conserved spatial distance between the TL and the 5′ end, further support shared NS5-binding mode across these viruses. The SLA of YFV shows a unique 3WJ and the measured distance between the TL and the 5′ end is longer than that observed for other MBFV and TBFV (Fig 4H). The divergent position of YFV within the MBFV group is consistent with unique SLA structural properties of this virus. Nevertheless, the SLA of YFV was found to be functional in the context of DENV and ZIKV.

Together our data indicate that productive viral replication requires not only conserved structures and specific sequences within individual SLA sub-elements, but also their coordinated 3D organization. Although certain SLA features have diverged in a group-specific manner, the combination of these elements maintains a conserved global architecture in pathogenic orthoflvivirus SLAs that enables heterologous NS5 recognition and viral RNA replication. Previous studies have shown that NS5 binding to SLA induces alterations in the RNA footprinting pattern at the 3’ of the BS, supporting conformational changes in the RNA far away from the proposed NS5 binding sites [46]. More recent studies, also suggested that upon RNP complex formation both NS5 domains and the SSL of the SLA undergo conformational rearrangement [49,70] highlighting the complexity of the SLA-NS5 interaction. The lack of structural information capturing the SLA-NS5 complex with the 3′ end of the RNA engaged in the polymerase template channel, which may require structural rearrangements of NS5, represents a key challenge for further understanding the initiation mechanism of viral RNA synthesis.

Binding of both, RdRp and the MTase, domains of NS5 to the TL and 5’ end of the SLA has been proposed to have a functional relevance [43,45] and a critical role was assigned to the linker region between these two domains [71]. Cooperative binding of the RdRp and the MTase to SLA has been also reported [41,43,49,72]. Nevertheless, during infection the two enzymatic activities act on different viral RNA molecules, thus it is still unclear the link between these two activities. In this regard, viral minus-strand RNA synthesis and methylation of the viral RNA are two events that take place at different stages of the viral RNA replication process. While minus-strand synthesis initiates at the 3’ end of the plus-strand genomic RNA, using a promoter SLA that is already capped and methylated, the process of cap methylation takes place at the 5’ end of a newly synthesized plus-strand RNA molecule. In this regard, the mechanism of initiation of the plus-strand RNA synthesis is still unclear. It has been recently reported that the 3’ end of the minus-strand forms an SLA-like structure (SLA’) that binds NS5 but is inactive for initiation of plus-strand RNA synthesis [59]. It is possible that the SLA of the plus strand also serves as promoter for new plus-strand synthesis by a process of trans initiation. Trans initiation in vitro by the DENV2 NS5 has been previously observed, using as promoter an SLA from one molecule to initiate at the 3’ end of a different molecule [31,33,36]. In this case, NS5 binding to a newly synthesized SLA would facilitate cap methylation. We conclude that viral RNA replication in infected cells requires multiple coordinated SLA-NS5 interactions, and that several key mechanistic aspects of this process remain unsolved.

Beyond RdRp and MTase activities, orthoflavivirus NS5 is involved in host immune evasion [73]. For instance, in the case of DENV, NS5 mediates STAT2 and ERC1 degradation [74,75]. Different innate immune environments in distinct hosts are expected to impose distinct selective pressures, leading to evolutionary divergence in NS5 across ecological groups. While the NS5 binding sites to SLA are essential for viral replication of all members of the genus, the NS5 regions that mediate immune evasion may undergo a host-driven evolutionary divergence. This divergence in NS5 sequence and structure could modulate protein conformations, that in turn, could affect SLA recognition in distinct ecological groups.

Since SLA is located within the 5’UTR, it is not constrained by protein-coding requirements and can therefore explore a broader structural and evolutionary landscape. We propose that most of the sequence and structural SLA ecological divergence observed arises from co-evolution with NS5 under host-specific selective pressures. However, we do not exclude the possibility that certain SLA features, such as divergence of the 3WJ, may also reflect evolutionary adaptations driven by ecological or functional constraints, contributing to SLA diversification across orthoflavivirus groups. Ecologically conserved sub-elements within SLAs may act as molecular signatures for human pathogenic orthoflaviviruses, providing a tool for the early recognition of emerging pathogenic viruses in natural reservoirs.

In summary, our work defines key structural and functional properties of an essential viral RNA element that is conserved among pathogenic orthoflaviviruses. The identification of small molecules that bind SLA and inhibit infection of multiple viruses provides an opportunity for developing antiviral strategies against known and emerging orthoflaviviruses.

## Materials and methods

### Databases

From the ICTV *Genus: Orthoflavivirus* webpage, we built a database containing 92 viruses (S1 Table). Within these viruses are included the orthoflavivirus species and the related, unclassified species, excepting the segmented flavi-like viruses. Additionally, we built an SLA database by selecting those viruses which 5’ sequences start in AG consisting in SLAs from 38 viruses (S2 Table).

### Sequence alignments

For RNA sequence alignment Clustal Omega [76] was used, while NS5 protein sequence alignment was obtained using MUSCLE [77]. Sequence logos were obtained using WebLogo3 web server [78]. From NS5 protein sequence alignment we built phylogenetic trees using IQ-TREE [79] and iTOL web servers [80].

### SLA 2D structure prediction and analysis

SLA secondary structure is available from experimental data only for DENV1, DENV2, DENV3, DENV4, ZIKV, WNV and YFV [47,48,81–83]. For the other viruses, the SLA secondary structure was predicted through RNAfold webserver from the Vienna RNA websuite [84] using the Turner 1999 model [85]. Webserver and model were chosen because of the similarity between the predicted and the experimental secondary structures of DENV1 to 4, ZIKV and YFV. Forna was used as a tool for 2D RNA visualization [86].

### Alignments considering 2D structures

To align multiple RNA sequences and predict a consensus secondary structure we used LocRNA 2.0 [87]. As input, we used the sequence and 2D structures predicted using RNAfold webserver with the Turner 1999 model. Secondary structure conservation was calculated for each position in the alignment. We evaluated whether the nucleotide was absent, it was found single or it was found double stranded and calculated a conservation index, defined as the frequency with which the state of that position matched the consensus structure.

### SLA 2D structure analysis

From the 2D structures and alignment, the following parameters were determined for each SLA:

*The stem length* was considered from the number of nucleotides single or double stranded in the 5’ side of the stem.

*The 4 nucleotide TL sequence* was determined from a TL sequence alignment. DENV2 and ZIKV TL were used as reference to define 4-nucleotides long TL sequences for each virus.

*Nucleotides in the 3WJ* were identified based on their alignment to the loop regions of the 3WJ in the consensus structure obtained from group-specific sequence–structure alignments.

### Molecular dynamics

The atomistic model for the SLA-NS5 RNP complex was derived from the Cryo-EM structure with PDB ID 8GZP. Internal regions that were unresolved due to high flexibility were modeled by homology using the SWISS-MODEL program [88] based on the NS5-DENV3 sequence reported in the structure. Similarly, for the SLA (nucleotides 1–69), two nucleotides from the side stem-loop were unresolved and were modeled using ModeRNA [89], a homology modeling program specific to RNA. Although the structure was resolved in the presence of the 5′ cap, the diphosphate guanosine and a Mg²⁺ cation in the MTase catalytic site, both were removed due to experimental evidence showing their presence does not affect SLA-NS5 interaction [90]. The two resolved Zn²⁺ cations were included in the structure.

The protein and RNA were modeled using the Amber ff14SB force field [91] with the ILDN correction for protein residues and updated dihedral parameters for RNA [92]. Parameters for the four amino acids (C778, C847, H712, and H714) that form one Zn2+ tetrahedral environments were obtained from the Zinc AMBER Force Field (ZAFF) [93]. Parameters for the other four amino acids (C446, C449, H441, and E437) coordinating the second Zn2+ were derived using the Metal Center Parameter Builder in Python: MCPB.py [94]. The interactions modelled by these parameters act as covalent-like bonds between the metal and the four coordinating amino acids. To obtain the latter Zn2+ parameters, equilibrium distances and angles between atoms were first calculated for the gas-phase system using ab initio methods. Then, force constants were obtained from minimization of an approximate model of the system without the metal (e.g., converting thiol (SH) to thiolate (SO⁻) in cysteine residues that coordinate the metal), using DFT at the B3LYP/6-31G* level. Finally, partial charges were assigned using the RESP method, implemented in Gaussian 16.

Protonation states of ionizable residues were determined based on estimated pKa values using PropKa 3.0 [95], an empirical pKa predictor for surface and buried residues. After inspecting the environment of each histidine, the following were assigned as HID: H215, H263, H441, H512, H712, H714, and H768; the rest were assigned as HIE. Using tLeap module [96] the system was solvated using approximately 45,000 TIP3P water molecules [97] placed in an octahedral box of 15 Å from the protein surface. A physiological salt concentration of 0.15 M was obtained by adding 123 Na+ and Cl- ions. Extra 53 Na+ were included to neutralize the system charge (-53 e).

Triplicate simulations were carried out using the PMEMD Cuda module of the Amber 22 package [96]. Each replica included the three pre-production stages (minimization, heating, and equilibration). At the beginning of the pre-production, water molecules and ions were energy-minimized with positional restraints (300 kcal/mol/Å²) on biomolecule atoms (protein and RNA). This involved 500 steps of steepest descent followed by 500 steps of conjugate gradient. Next, restraints were removed, and 500 more steps of steepest descent and 2,500 steps of conjugate gradient were performed. After energy minimization, the system was heated to 300 K and 1 bar in three stages, all at constant volume (NVT ensemble): first, temperature ramped from 0 to 100 K over 5 ps with strong restraints (100 kcal/mol/Å²) on biomolecule atoms, relaxing only solvent and ions; second, the restraints were reduced to 25 kcal/mol/Å², and the temperature raised to 200 K over 10 ps; and third, temperature increased from 200 K to 300 K with 10 kcal/mol/Å² restraints over 100 ps. Finally, the constant pressure equilibration was performed in two steps (NPT ensemble): first, 100 ps at 300 K and 1 bar with 10 kcal/mol/Å² restraints; and second, 10,000 ps at 300 K and 1 bar without restraints. A 1-fs time step was used during heating; 2 fs during equilibration. Temperature was controlled with the Langevin thermostat (collision frequency: 1 ps ⁻ ¹) [98], and pressure, for NPT equilibration, was maintained with the Berendsen barostat (relaxation time: 2 ps) [99]. The SHAKE algorithm [100] constrained bonds involving nonpolar hydrogens and a 10 Å cutoff was used for nonbonded interactions. Three independent 2-μs production runs were performed for each system using the NVT ensemble and a 2-fs time step. The first 0.5 μs of each trajectory was discarded as additional equilibration.

### SLA 3D structure prediction

3D prediction was performed using RNAComposer [101]. Predicted secondary was used and 5 different models were obtained for each SLA. To visualize 3D structures, VMD and ChimeraX were used [102,103].

### SLA 3D structure analyses

We used DSSR [104] to analyze the 3D structures. TL-5’ end distances were calculated using the origin of the frame of reference for the two bases as defined in the DSSR software. The second position of the TL was chosen as the TL reference, identified by performing floor division of the TS hairpin length and subtracting 1 for each SLA.

The *TL RMSD value* was calculated for every 3D model with respect to the NS5-bound DV4 structure (pdb 8gzp). All RMSD values were calculated after aligning TL to the same reference structure and only the nucleic acid backbone atoms (OP2, OP1, P, O5’, C5’, C4’, C3’, O3’) were included.

*Coaxial stacking* was identified with DSSR as a helix of at least 10bps, with the first and last pairs being separated by no more than 8 nts (generous criteria for TL or SSL). A co-axial stacking of the side stem (SS) with the top stem (TS) observed in the SLA-NS5 complex structures is stable throughout all of three independent microsecond MD simulations (97% of conformations).

### Construction of chimeric and sub-chimeric viruses

For constructing the chimeric viruses containing mutations in the SLA, we modified DENV2 [51,105] and ZIKV infectious clones [50]. Mutations were introduced in the ICRep DENV2 replacing the MluI-NotI fragment with the respective fragments derived from PCRs (S3 Table) or overlapping PCRs (S4 Table) containing the desired sequence. In the case of the ZIKV, mutations were introduced in the IC ZIKV replacing the MluI-XhoI fragment and in the ICRep ZIKV replacing the MluI-AvrII fragment with fragments derived from PCRs (S3 Table) or overlapping PCRs (S4 Table) containing the desired sequence. S5 Table contains sequence from the primers used.

The ligation products were transformed into XL1-Blue bacteria and several clones for each mutant were obtained. The resulting plasmids were sequenced, and the positive clones were used for in vitro RNA transcription.

### In vitro transcription and transfection

Plasmids containing the wild type and chimeric viruses were linearized with XbaI for DENV2 and KpnI for ZIKV; and used for *in vitro* transcription using T7 RNA polymerase (Ambion) in the presence of m7GpppA cap analog (NEB). We modified the last two nucleotides of the T7 promoter (GG by GA) so that the transcripts initiate with the authentic m7GpppAGU. RNA quantification was assessed using a Qubit4 (Invitrogen) and RNA integrity was confirmed on 1% agarose gels. RNA transcripts were transfected into BHK and C6/36HT cells using Lipofectamine 2000 and Opti-MEM media (Invitrogen). For 24 well cell culture plates, 50 ng of viral RNA per well were used.

### Cell culture

Transfections and infections were performed using the following cell lines. C6/36HT cells (ATCC CRL-1660), an *Aedes albopictus* cell line adapted to grow at 33°C, were cultured in Leibovitz’s L-15 medium supplemented with 10% fetal bovine serum (FBS), 100 U/ml of penicillin, 100 μg/ml of streptomycin, 0.3% tryptose phosphate broth, 0.02% glutamine, 1% minimal essential medium (MEM) nonessential amino acid solution, and 0.25 μg/ml of amphotericin B (Fungizone). Baby hamster kidney cells (BHK, ATCC, CCL-10) were cultured in Minimum Essential Medium α (MEMα) (Gibco, Thermo Fisher Scientific) supplemented with 10% fetal bovine serum (FBS) (Gibco, Thermo Fisher Scientific) and 100 U/ml penicillin-streptomycin in 100mm cell culture plates and incubated at 37°C with 5% CO2. A549 cells (human lung adenocarcinoma epithelial cell line, ATCC, CCL- 185) were cultured in Dulbecco’s modified Eagle’s medium with nutrient mixture F-12 (DMEM-F-12) supplemented with 10% fetal bovine serum, 100 U/ml of penicillin, and 100 μg/ml of streptomycin.

### Lucifirese activity and statistical analysis of DENVRep replication

Renilla luciferase assays were performed using Renilla luciferase assay system kit (Promega). Replication of reporter viruses was classified integrating the temporal behavior of the luciferase signal and comparisons with the non-replicative control (NS5 mut).

Chimeric viruses were classified as replicative, suboptimal and non-replicative. A chimera was defined as replicative when the luminescence signal was not significantly different from WT at every time point, as suboptimal when a statistically significant increase in signal relative to non-replicative control was detected at 72 hours post transfection, and as non-replicative when the luciferase signal was not significantly different from the non-replicative control. Statistical comparisons were performed using two-tailed Welch’s t-tests, taking into account three biological replicates.

### Immunofluorescence assay

BHK-21 cells were grown in 24-well cell culture plates containing 12mm glass coverslips. At the corresponding times post transfection or infection, the coverslips were collected, and cells were fixed with methanol for 15 min at –20°C. The coverslips were blocked using gelatin 0.2% (Sigma) in PBS and incubated with mouse monoclonal anti-E antibody (E18) was used diluted 1/500 in blocking solution for DENV2 and with rabbit anti-NS3 polyclonal antibody diluted 1/500 in blocking solution for ZIKV. Goat anti-rabbit antibody Alexa Fluor 488 conjugate (Thermo Fisher Scientific) was employed to detect the primary antibody and DAPI (Thermo Fisher Scientific) was added to visualize the nuclei. Images were obtained using Axio Observer 3 (Zeiss) inverted fluorescence microscope, with 10x objective.

### Retrotranscription

Viral RNAs were extracted from supernatants of transfected cell using TRIzol (Invitrogen). Purified RNAs were used for reverse transcription-PCRs (RT-PCRs) in duplicates with primers specifically designed to amplify the first 500 nucleotides of ZIKV and DENV2 genome ends, using SuperScript III reverse transcriptase (Invitrogen) and Platinum Pfx DNA polymerase (Invitrogen).

### RT-qPCR

For qRT-PCR of viral RNA, supernatant RNA samples were used for reverse transcription as previously described. Reactions were performed in duplicates in 96-well plates using 2 μl of the RT reaction mixture as the template, 5 μl of FastStart SYBR green Master 2 × mix (Roche), a 300 nM concentration of each primer, and RNase-free water to 10 μl. For DENV2, the primers AVG1117 (5′ACAAGTCGAACAACCTGGTCCAT3′) and AVG1118 (5′GCCGCACCATTGGTCTTCTC3′) were targeted to amplify nucleotides 9937 to 10113 within the NS5 coding sequence. For ZIKV, the primers AVG2098 (5′GCCGCCACCAAGATGAACTGATTG3′) and AVG2099 (5′GCAGTCTCCCGGATGCTCCATC3′) were targeted to amplify nucleotides 9854 to 9927 within the NS5 coding sequence.

### Electrophoretic Mobility Shift Assays

To obtain RNAs, first a DNA template was prepared by PCR amplification. The nucleotide sequences of the primers used and the templates for the PCR for each synthetic RNA are detailed in S6 Table. Next, in vitro transcription was performed on the templates using T7 RNA polymerase (180 min, 37°C) and treated with RNase-free DNase I to remove templates. RNA products were purified by native polyacrylamide gel electrophoresis, followed by excision of the corresponding bands and elution. Final product quantification was assessed using a Qubit4 Fluorometer (Invitrogen), and integrity was confirmed on 2% agarose gels. The synthetic RNA sequences are specified in S6 Table.

For protein expression, a pQE-30 plasmid containing the sequence of the full length NS5 protein as used before [90]. NS5 was expressed in *Escherichia coli* Rosetta (pLacI) overnight at 18°C after induction with 100 μM isopropyl-β-d-thiogalactopyranoside (IPTG). Lysis of the cells was carried out by sonicating using in binding buffer (50 mM sodium phosphate, pH 7.5, 600 mM NaCl, 5 mM MgCl_2_, and 10% glycerol) in the presence of 0,2% v/v β-mercaptoethanol, 1% Triton X-100, 12 µg/ml DNase I, 1 mM PMSF and 1% v/v protease inhibitor cocktail (Sigma). After centrifugation, the supernatant was loaded on a His-Trap nickel Sepharose affinity column (GE Healthcare) and washed twice with binding buffer plus 80 mM imidazole, and the proteins were eluted with binding buffer containing 300 mM imidazole. Sample buffer was changed to HPLC buffer (10 mM Tris-HCl buffer, pH 7.5, 300 mM NaCl, 5 mM MgCl_2_, 10% glycerol, and 1 mM dithiothreitol) using Amicon Ultra centrifugal filters, 10 kDa MWCO (Merck Millipore). The protein was further purified by size exclusion chromatography using a Superdex 200 column (Cytiva). The protein was concentrated using Amicon Ultra centrifugal filter, 50 kDa MWCO (Merck Millipore) and stored at −20°C in HPLC buffer containing 40% glycerol.

For EMSA, the binding reaction mixture contained 5 mM HEPES (pH 7.9), 20 mM NaCl, 2 mM MgCl_2_, 3.8% glycerol, 10 nM RNA probe, and increasing concentrations of the protein. RNA-NS5 complexes were analyzed by electrophoresis through native 5% polyacrylamide gels supplemented with 5% glycerol. Gels were prerun for 10 min at 80 V, and then 20 μl of sample was loaded, and electrophoresis was allowed to proceed for 1 h at 4°C at a constant voltage of 60 V. Gels were stained with SYBR Gold (Thermo Fisher Scientific) and imaged with a transilluminator.

### Screening for RNA binders

*Z-Score Determination*. RNA was annealed at 95 °C for 5 minutes and snap-cooled on ice for 30 minutes. After the RNA was annealed, a 0.085 μM DENV SLA and 0.5 μM TO-PRO-1 solution was made in buffer (5 mM HEPES (pH 7.9), 25 mM KCl, 2 mM MgCl2) and incubated on a rocker for 30 minutes. 5 μL of 10 or 25 μM neomycin or buffer followed by 5 μL of the RNA-dye solution was added to a black 384-well plate. The plate was centrifuged for 1 minute and left to incubate for 30 minutes. After incubation, the plate was read on a CLARIOstar plate reader (BMG Labtech) (excitation = 492 nm; emission = 575). The Z’-Score was calculated based on literature standards [106].

*Small Molecule Screen.* RNA was annealed at 95 °C for 5 minutes and snap-cooled on ice for 30 minutes. 10 nL of small molecules were plated in black 384-well plates using an Echo 550 Acoustic liquid handler (Labcyte). 0.085 μM DENV SLA and 0.5 μM TO-PRO-1 solutions were made using buffer (5 mM HEPES (pH 7.9), 25 mM KCl, 2 mM MgCl2) and then the solutions were rocked and incubated for 30 minutes. 10 μL of the RNA-dye solution was transferred to the plate using a ThermoFisher liquid dispenser and then the plates were shaken, centrifuged, and left to incubate for 30 minutes. After incubation, the plate was read on a CLARIOstar plate reader (BMG Labtech) (excitation = 492 nm; emission = 575). The % displacement was calculated using the equation below where F is the fluorescence of the RNA-dye complex with small molecules and F0 is the fluorescence of the RNA-dye complex without small molecule. Screening was performed in triplicate. Small molecules that showed greater than or equal to 20% FID in at least 2/3 replicates and that averaged greater than or equal to 20% FID over the three replicates were considered hits.


$$\begin{array}{c} \%\text{ FID}=\text{100}-(\text{100}*\left(\frac{F}{F0}\right)) \end{array}$$

### Viral stocks

For obtaining DENV2 and ZIKV viral stocks, supernatants of transfected C6/36 HT cells were collected at 7dpt, centrifugated at 1000 rpm for 5 minutes and subsequently titrated. For YFV, RNA corresponding to the 17D virus [107] was transfected into BHK cells and supernatant was collected at 3 dpt, centrifugated at 1000 rpm for 5 minutes and subsequently titrated. Finally, for LGTV, RNA corresponding to the TP21 virus [63] was transfected into BHK cells and supernatant was collected at 3 dpt, centrifugated at 1000 rpm for 5 minutes and subsequently titrated.

### Inhibition infection assays

A549 cells were infected with viral stocks diluted in F-12 medium. DENV2 and LGTV infections were performed at a multiplicity of infection (MOI) of 0.1, whereas YFV and ZIKV infections were carried out at an MOI of 0.01. At 1h post-infection (hpi), the inoculum was removed and replaced with F-12 medium containing the indicated concentration of small molecule, supplemented with 1% DMSO and 2% fetal bovine serum. Infected cultures were harvested at 24 hpi.

### Cell viability assay

The viability of A549 cells following inhibitor treatment was determined by measuring cellular ATP levels, indicative of metabolic activity. This was achieved using the CellTiter-Glo Luminescent Cell Viability Assay kit (Promega), following the manufacturer’s instructions.

### Plaque assays

BHK-21 cells were seeded in 24-well cell culture plates and grown overnight. Transfected or infected BHK-21 cells supernatants were serially diluted and 200 µl of the inoculum was used to infect BHK-21 monolayers for 1 hour at 37°C. Afterwards, 1 ml of overlay medium (MEMα, 0.8% methyl cellulose, supplemented with 5% FBS) was added to each well. Cells were fixed 7-day post-infection (dpi) with 10% formaldehyde and stained with crystal violet.

## Supporting information

S1 Fig Impact of third nucleotide identity of the viral genome on RNA replication of DENVRep chimeras carrying SLAs from different MBFV. Replication of DENVRep chimeras carrying SLAs from SLEV, JEV, ILHV, USUV and ROCV are shown. Chimeric viruses containing the native A at the third position (5’AGA) or U (5’AGU) are indicated in each case. Normalized luciferase activity as a function of time in BHK cells transfected with RNAs corresponding to the DENVRep WT, chimeras and NS5 Mut, as indicated on the top. Comparison between each chimera carrying 5’AGA or 5’AGU at 72 h were performed using two-tailed Welch’s t-tests. * indicates adjusted p-value <0.05.
(EPS)

S2 Fig Interactions between G32 of the SLA top loop and either R770 (blue) or K841 (orange) of the NS5 RdRp thumb region. Probability density distributions of the distances computed from aggregated molecular dynamics trajectories (3 μs total from three replicas). Kernel density estimates are shown for each interaction (bandwidth determined using Scott’s rule). Dashed vertical lines indicate the corresponding distances in the initial structure (PDB 8gzp), highlighting deviations from the initial contact geometry during the simulations. Distances were calculated using the carbonyl oxygen (O6) of the guanine base (G32), the central carbon atom (CZ) of the R770 guanidinium group and the nitrogen atom (NZ) of the ε-ammonium group of K841. Data visualized using Seaborn (1).
(EPS)

S1 Table List of the orthoflaviviruses used in this study. (DOCX)

S2 Table List of SLA sequences used with their secondary structure in dot-bracket notation. (DOCX)

S3 Table PCR designs to construct chimeric viruses. (DOCX)

S4 Table Oligonucleotide used for overlapping PCRs to construct chimeric viruses. (DOCX)

S5 Table Primer sequences. (DOCX)

S6 Table Primer sequences to construct SLA RNAs for in vitro binding assays. (DOCX)
