# Supplementary material for: The RNA promoter for pathogenic orthoflaviviruses replication is universal and serves as target for viral inhibition
Source: PLoS Pathog. 2026 May 18;22(5):e1014233. doi: 10.1371/journal.ppat.1014233 (PMC13211259; doi:10.1371/journal.ppat.1014233)
Supplement: S1 Table — (DOCX) [file ppat.1014233.s003.docx]

***S1 Table:*** *List of the orthoflaviviruses used in this study*

| **Virus** | **Short name** | **Ecologic group** | **Accession number** |
| --- | --- | --- | --- |
| Aedes flavivirus | AeFV | ISFV | NC_012932.1 |
| Aedes galloisi flavivirus | AGFV | ISFV | AB639347.1 |
| Anopheles flavivirus | AnFV | ISFV | KX148546 |
| Apoi virus | APOIV | NKVF | NC_003676.1 |
| Aroa virus | AROV | MBFV | NC_009026.2 |
| Bagaza virus | BAGV | MBFV | NC_012534.1 |
| Banzi virus | BANV | MBFV | NC_043110.1 |
| Barkedji virus | BJV | ISFV | KC496020 |
| Bouboui virus | BOUV | MBFV | NC_033693.1 |
| Bukalasa bat virus | BUBV | NKVF | OL709419.1 |
| Cacipacore virus | CPCV | MBFV | NC_026623.1 |
| Calbertado virus | CLBOV | ISFV | KX669682.1 |
| Carey Island virus | CIV | NKVF | NC_043112.1 |
| Cell fusing agent virus | CFAV | ISFV | LR596014.1 |
| Chaoyang virus | CHOV | ISFV | NC_017086.1 |
| Cowbone Ridge virus | CRV | NKVF | AF297461.1 |
| Cuacua virus | CuCuV | ISFV | KX245154 |
| Culex flavivirus | CxFV | ISFV | NC_008604.2 |
| Culex Theileri Flavivirus | CxThFV | ISFV | HE574574.1 |
| Culiseta flavivirus | CsFV | ISFV | KT599442 |
| Dakar bat virus | DBV | NKVF | OL709418 |
| Dengue virus 1 | DENV1 | MBFV | AY145121.1 |
| Dengue virus 2 | DENV2 | MBFV | U87411.1 |
| Dengue virus 3 | DENV3 | MBFV | AY648961.1 |
| Dengue virus 4 | DENV4 | MBFV | AY648301.1 |
| Donggang virus | DONV | ISFV | NC_016997.1 |
| Edge Hill virus | EHV | MBFV | NC_030289.1 |
| Entebbe bat virus | ENTV | NKVF | NC_008718.1 |
| Fitzroy River virus | FRV | MBFV | KM361634 |
| Gadgets Gully virus | GGYV | TBFV | MN830233.1 |
| Hanko virus | HAFV | ISFV | ON949929.1 |
| Ilheus virus | ILHV | MBFV | OP947882.1 |
| Ilomantsi virus | ILOV | NKVF | KC692067 |
| Israel turkey meningoencephalomyelitis virus | ITV | MBFV | KC734551.1 |
| Japanese encephalitis virus | JEV | MBFV | HM596272.1 |
| Jugra virus | JUGV | MBFV | NC_033699.1 |
| Jutiapa virus | JUTV | NKVF | KJ469371.1 |
| Kadam virus | KADV | TB | NC_033724.1 |
| Kamiti River virus | KRV | ISFV | NC_005064.1 |
| Kampung Karu virus | KPKV | NKVF | NC_040788.1 |
| Kedougou virus | KEDV | MBFV | MZ218098.1 |
| Kokobera virus | KOKV | MBFV | MZ358852.1 |
| Koutango virus | KOUTV | MBFV | OQ067500.1 |
| Kyasanur Forest disease virus | KFDV | TBFV | HM055369.1 |
| La Tina virus | LTNV | NKVF | KY320649.1 |
| Lammi virus | LAMV | ISFV | FJ606789.2 |
| Langat virus | LGTV | TBFV | NC_003690.1 |
| Long Pine Key virus | LPKV | NKVF | MZ090957.1 |
| Louping ill virus | LIV | TBFV | KP144331.1 |
| Marisma mosquito virus | MMV | NKVF | MF139576 |
| Meaban virus | MEAV | TB | NC_033721.1 |
| Mercadeo virus | MECDV | ISFV | NC_027819.1 |
| Modoc virus | MODV | NKVF | NC_003635.1 |
| Montana myotis leukoencephalitis virus | MMLV | NKVF | NC_004119.1 |
| Murray Valley encephalitis virus | MVEV | MBFV | KF751871.1 |
| Nakiwogo virus | NAKV | ISFV | GQ165809.2 |
| Nanay virus | NANV | ISFV | NC_040610.1 |
| Ngoye virus | NGOV | NKVF | DQ400858.1 |
| Nhumirim virus | NHUV | ISFV | NC_024017.1 |
| Nienokoue virus | NiFV | ISFV | JQ957875.2 |
| Nounane virus | NOUV | ISFV | NC_033715.1 |
| Ntaya virus | NTAV | MBFV | NC_018705.3 |
| Omsk hemorrhagic fever virus | OHFV | TBFV | NC_005062.1 |
| Palm Creek virus | PCFV | ISFV | KC505248.1 |
| Paraiso Escondido virus | EPEV | ISFV | NC_027999.1 |
| Parramatta River virus | PaRV | ISFV | NC_027817.1 |
| Phnom Penh bat virus | PPBV | NKFV | NC_034007.1 |
| Powassan virus | POWV | TBFV | NC_003687.1 |
| Quang Binh virus | QBV | ISFV | AB981186.1 |
| Rio Bravo virus | RBV | NKVF | JQ582840.1 |
| Royal Farm virus | RFV | TBFV | DQ235149.1 |
| Saboya virus | SABV | MBFV | NC_033697.1 |
| Saint Louis encephalitis virus | SLEV | MBFV | NC_007580.2 |
| Sal Vieja virus | SVV | NKVF | AF297460.1 |
| San Perlita virus | SPV | NKFV | AF013402.1 |
| Saumarez Reef virus | SREV | TBFV | MW959115.1 |
| Sepik virus | SEPV | MBFV | NC_008719.1 |
| Spondweni virus | SPOV | MBFV | DQ859064.1 |
| Tamana bat virus | TABV | NKVF | MZ229974.1 |
| Tembusu virus | TMUV | MBFV | NC_015843.2 |
| T'Ho virus | THOFV | MBFV | NC_034151.1 |
| Tick-borne encephalitis virus | TBEV | TBFV | NC_001672.1 |
| Tyuleniy virus | TYUV | TBFV | KT224356.1 |
| Uganda S virus | UGSV | MBFV | NC_033698.1 |
| Usutu virus | USUV | MBFV | OP007489.1 |
| Wesselsbron virus | WESSV | MBFV | NC_012735.1 |
| West Nile virus | WNV | MBFV | AF404756.1 |
| Xishuangbanna aedes flavivirus | XFV | ISFV | NC_034017.1 |
| Yamadai flavivirus | YDFV | ISFV | MT254448.1 |
| Yaoundé virus | YAOV | MBFV | EU082199.2 |
| Yellow fever virus | YFV | MBFV | X03700.1 |
| Yokose virus | YOKV | NKVF | NC_005039.1 |
| Zika virus | ZIKV | MBFV | MT636065.1 |
