# Supplementary material for: The RNA promoter for pathogenic orthoflaviviruses replication is universal and serves as target for viral inhibition
Source: PLoS Pathog. 2026 May 18;22(5):e1014233. doi: 10.1371/journal.ppat.1014233 (PMC13211259; doi:10.1371/journal.ppat.1014233)
Supplement: S2 Table — (DOCX) [file ppat.1014233.s004.docx]

***S2 Table:*** *List of SLA sequences used with their secondary structure in dot-bracket notation.*

| **Virus** | **SLA sequence** | **SLA structure** |
| --- | --- | --- |
| AeFV | AGUUUUUAAAAACUUCUGUGAAGGUCACAUCCCUAGUUGGAUUACGCCGCUUUUGAAAAAUCUAGAAGUUGUUUGGAAA | ..((((((((((((((((.((.(((...((((......))))...)))....((....)))))))))))).)))))))) |
| AROV | AGUAUUUCUUCUGCGUGAGACCAUUGCGACAGUUCGUACCGGUGAGUUUUGACUUAACGCAGUGAGAAA | ....(((((((((((((((((((((((((....)))))...))).)))))......)))))).)))))) |
| BAGV | AGAAGUUCAUCUGUGUGAACUUUGUGAUUGACAGCUCAACACAAGUGCGGACUACCGUAAACACAGUUUGAAC | ....(((((.((((((....((((((.((((....))))))))))(((((....))))).))))))..))))) |
| BJV | AGUUUGUGCUGUACGUGCACUGGGAAAACACAGUUGGUAUUUCUGGGUGGAAACAUAACGUAUUCGCGCA | ....(((((.((((((...(((((((.((.......)).)))))))(((....))).))))))..))))) |
| CFAV | AGUUUAUAAAAACUUCGGCUUGGCUACACCACAGUUUUGGUUACGCUUAUACUUUCAAAGCUUAAGUUGUUUUUA | ..........(((((.((((((((...((((......))))...)))..........))))).)))))....... |
| CLBOV | aGUUUUAAAGAGUUUACCGCUGUUUUCGAAUCAGUUUAUCGAAGGCGUUCUAUAGCGCAAGCUCGAGAUAAAAG | .........((((((..((((((((((((.........)))))))).......)))).)))))).......... |
| CxFV | AGUUUUUAAAAACUUCGGCUUGGUUACACCGCAGAUUGGUUACACCUACACAAGGCUUGAGUUGUUUAUAA | ..........(((((.((((((((...((((.....))))...)))......))))).)))))........ |
| DENV1 | AGUUGUUAGUCUACGUGGACCGACAAGAACAGUUUCGAAUCGGAAGCUUGCUUAACGUAGUUCUAACAG | ...((((((.((((((.((.((........((((((......)))))))).)).))))))..)))))). |
| DENV2 | AGUUGUUAGUCUACGUGGACCGACAAAGACAGAUUCUUUGAGGGAGCUAAGCUCAACGUAGUUCUAACAGU | ..(((((((.((((((...((..((((((.....)))))).))((((...)))).))))))..))))))). |
| DENV3 | AGUUGUUAGUCUACGUGGACCGACAAGAACAGUUUCGACUCGGAAGCUUGCUUAACGUAGUACUGACAG | ...(((((((((((((.((.((........((((((......)))))))).)).)))))).))))))). |
| DENV4 | AGUUGUUAGUCUGUGUGGACCGACAAGGACAGUUCCAAAUCGGAAGCUUGCUUAACACAGUUCUAACAG | ...((((((.((((((((.((((...(((....)))...))))...))......))))))..)))))). |
| ENTV | AGUAAAUUUUGCGUGCUAGUCGCUUGGCGUUAGUCCGUGAAGUGAGUUUUUGGAUACAUUGUACCAGAGAUUAACACGUUGAAAUU | ......(((.(((((....((((((.(((......))).))))))((((((((.(((...)))))))))))...))))).)))... |
| EPEV | AGUAAAUCUUGCAUGUGGAUUAGUGAGCACUAGUUUGCGCAUUGAAACAAAUUGUGGAUUAACUUUUGUUAACGUGUUUGGAUU | ....(((((.((((((...((((((.(((......))).))))))((((((..((......)).)))))).))))))..))))) |
| ILHV | AGAAAUUCACCUGUGUGAAUUUCACUAACCGUUUUAGUGGAGAGAACUUUUGUUUAACACAGUCUGAAUAGUU | ....(((((.((((((....((((((((.....))))))))..((((....)))).))))))..))))).... |
| JEV | AGAAGUUUAUCUGUGUGAACUUCUUGACUUAGUAUCGUUGAGAGGAAUCGAGAGAUUAGUGCAGUUUAAAC | ....(((((.((((.....(((((((((........)))))))))((((....))))...))))..))))) |
| KFDV | AGAUUUUUUUGCACGUGAGUGCUCUCGUUUCAGGCAACGUGAGUGGCGCUUUGUUGGUAUUUCCUUGGUGGGAAAGUGUUGAAGCGUUAACGUGUUGAGGAAA | ...((((((.((((((....((((.((((......)))).))))(((((((((......((((((....))))))....))))))))).)))))).)))))). |
| KOKV | AGAUGUUCACCUGUGUGAACUAACCAGACAGAUCAAAGUUAGGUGGAAACAUAACACAGUGUGAGCA | ...(((((((((((((......(((.(((........))).))).........)))))).)))))))................ |
| KOUTV | AGUAGUUCACCUGCGUGGACUGGCAAACUCAGCAUCGUUUGCGAGAAUUUUAACGAAUUAACACAGUGUGAAUUG | ..(((((((((((.((...((.((((((........)))))).))(((((....))))).)).))).)))))))) |
| KRV | AGUUUUUGAAAACUUCUGUGAAUGUUUAUAUCCUUAGUCGGAUCGAGCUAAAUUUUAAAUCAAAGGAGUUGUUCGGAAA | ..(((((((((((((((.(((..((((..((((......)))).))))...........))).))))))).)))))))) |
| LAMV | AGUAUAUUCUACGUGUGCGUUUUGUGACAUAGUUAGUCAGAAGAGUAUUUUGAAGUAUAACACGUUUGAAUA | ....(((((.((((((...((((.((((.......)))).))))(((((....))))).))))))..))))) |
| LGTV | AGAUUUUCUUGCGCGUGCAUGCGUGUGCUUCAGACAGCCCAGGCAGCGACUGUGAUUGUGGAUAUUCUUUCUGCAAGUUUUGUCGUGAACGUGUUGAGAAAA | ...(((((((((((((...(((.((.(((......))).)).)))(((((.....(((((((.......))))))).....)))))..))))).)))))))) |
| LIV | AGAUUUUCUUGCACGUGCGUGCUAUAGCUUCGGACAGCUUUGGCAGCGGCAGGUUUGAAAGAGACACUUUUUCACUUUCAUCAGUCGUGAACGUGUUGAGAAAA | ...(((((((((((((...(((((.((((......)))).)))))(((((.(((.((((((((...)))))))).....))).)))))..))))).)))))))) |
| MMLV | AGUUGGUUUUGCCGGCUACAACGAUCCUCCGUAGGAAGCGUUGGUGUCUUGGACAUUGCCGAGUUGAAACCU | ....((((((((((((..(((((.((((....))))..)))))(((((...))))).)))).)).)))))). |
| MODV | AGUUGAUCCUGCCAGCGGUGGGUCGCUACUGUUUCGCGAACCAGUCGUUUUGACAGUUGGUUGGGAUCAA | ..((((((((((((((..(((.((((.........)))).)))(((.....))).))))).))))))))) |
| MVEV | AGACGUUCAUCUGCGUGAGCUUUCGAUCUCAGUAUUGUUUGAAAGGAUCAUUUUGAUUAACGCGGUUUGAACAGU | ....(((((.((((((...(((((((............)))))))(((((...))))).))))))..)))))... |
| NHUV | AGUUUGUGCUGUACGUGCACUGGAAGUACAUAGUUGGUAUAACGGGUAGAAAUAUAACGUAUUCGCGCA | ....(((((.((((((...(((...((((.......))))..)))(((....))).))))))..))))) |
| NTAV | AGAAGUUCAUCUGUGUGAACUUCGUGAUUGACAGCUCAACACGAGUGCGGGCAACCGUAAACACAGUUUGAAC | ....(((((.((((((....((((((.((((....))))))))))(((((....))))).))))))..))))) |
| OHFV | AGAUUUUCUUGCACGUGCGUGCGCUUGCUUCAGACAGCAAUAGCAGCGGCAGGGUUGGUGGAAGGAAUUGCCCGCAUCAGCCAGUCGUGAACGUGUUGAGAAAA | ...(((((((((((((...(((..(((((......)))))..)))(((((..((((((((...((......)).)))))))).)))))..))))).)))))))) |
| PaRV | AGUUUUUAAAAGUUAACCCGUGGUUUUACCCUAGUUUCGGUGAAUUUGCCAAUUUUACGGAAUAACUGUUUGAAAG | ..(((((((((((((..(((((((((((((........))))))...)))......))))..))))).)))))))) |
| POWV | AGAUUUUCUUGCACGUGUGUGCGGGUGCUUUAGUCAGUGUCCGCAGCGUUCUGUUGAACGUGAGUGUGUUGAGAAA | ...(((((..(((((....((((((..((......))..))))))((((((....))))))...))))).))))). |
| SLEV | AGAUGUUCGCGUCGGUGAGCGGAGAGGAAACAGAUUUCCUUUUUGGAGGAUAAUAACUUAACUUGACUGCGAACA | ...(((((((((((((...(((((((((((....)))))))))))(((........))).)).)))).))))))) |
| TBEV | AGAUUUUCUUGCACGUGCAUGCGUUUGCUUCGGACAGCAUUAGCAGCGGUUGGUUUGAAAGAGAUAUUCUUUUGUUUCUACCAGUCGUGAACGUGUUGAGAAAA | ...(((((((((((((...(((...((((......))))...)))(((((((((..((((.(((......))).)))).)))))))))..))))).)))))))) |
| TMUV | AGAAGUUCGCCUGUGUGAACUUAUUCCAAACAGCUUUUGGAGUAGUGCGUGUGAACGUAAACACAGUUUGAAC | ....(((((.((((((....((((((((((.....))))))))))(((((....))))).))))))..))))) |
| USUV | AGAUGUUGGCCUGUGUGAGCUCUACUACUUAGUAUUGUUUUUGGAGGAUCGUGAGAUUAACACAGUGCCGGCAGU | ...(((((((((((((...(((((..((........))...)))))((((....)))).)))))).))))))).. |
| WNV | AGUAGUUCGCCUGUGUGAGCUGACAAACUUAGUAGUGUUUGUGAGGAUUAACAACAAUUAACACAGUGCGAGCUG | ..((((((((((((((...((.((((((........)))))).))...............)))))).)))))))) |
| YFV | AGUAAAUCCUGUGUGCUAAUUGAGGUGCAUUGGUCUGCAAAUCGAGUUGCUAGGCAAUAAACACAUUUGGAUU | ....(((((.(((((....((((..((((......))))..))))(((((...)))))...)))))..))))) |
| YOKV | AGUAAAUUUUGCGUGCUAGUCGCUGAGCGUCAGACCGCAAAGUGAGUUUUUAGUGAUCUAAAGUGAGGAGUUAUUCUUACUGUCAUCAAACACUACAAAUAAACACGUUG | ..........(((((....(((((..(((......)))..)))))((((.(((((......((((((((....)))))))).........))))).))))...))))).. |
| ZIKV | AGUUGUUGAUCUGUGUGAAUCAGACUGCGACAGUUCGAGUUUGAAGCGAAAGCUAGCAACAGUAUCAACA | ...(((((((((((((...(((((((.(((....))))))))))(((....))).)).)))).))))))) |
