# Supplementary material for: The RNA promoter for pathogenic orthoflaviviruses replication is universal and serves as target for viral inhibition
Source: PLoS Pathog. 2026 May 18;22(5):e1014233. doi: 10.1371/journal.ppat.1014233 (PMC13211259; doi:10.1371/journal.ppat.1014233)
Supplement: S3 Table — (DOCX) [file ppat.1014233.s005.docx]

***S3 Table:*** *PCR designs to construct chimeric viruses.*

| **Viral backbone** | **Chimeric virus** | **Template** | **Forward primer** | **Reverse primer** |
| --- | --- | --- | --- | --- |
| DENV2 | SLA SLEV (5’ AGU) | ICRep DENV2 + SLA SLEV | 3247 | 422 |
|  | SLA ILHV (5’ AGU) | ICRep DENV2 + SLA ILHV | 3340 | 422 |
|  | SLA ROCV (5’ AGU) | ICRep DENV2 +SLA ROCV | 3340 | 422 |
|  | SLA JEV (5’ AGU) | ICRep DENV2 + SLA JEV | 3339 | 422 |
|  | SLA USUV (5’ AGU) | ICRep DENV2 + SLA USUV | 3341 | 422 |
| ZIKV | TL UGGU | ICRep ZIKV | 3124 | 2738 |
|  | TL CGGA | ICRep ZIKV | 3125 | 2738 |
|  | TL UAGU | ICRep ZIKV | 2763 | 2738 |
|  | TL UCAG | ICRep ZIKV | 2762 | 2738 |
|  | SSL YFV | ICRep ZIKV | 3128 | 2738 |
|  | 3WJ YFV | ICRep ZIKV | 3010 | 2738 |
|  | 3WJ TBEV | ICRep ZIKV | 3332 | 2738 |
|  | SLA NHUV-R | ICRep ZIKV + SLA NHUV | 2983 | 2738 |
