# Supplementary material for: The RNA promoter for pathogenic orthoflaviviruses replication is universal and serves as target for viral inhibition
Source: PLoS Pathog. 2026 May 18;22(5):e1014233. doi: 10.1371/journal.ppat.1014233 (PMC13211259; doi:10.1371/journal.ppat.1014233)
Supplement: S4 Table — (DOCX) [file ppat.1014233.s006.docx]

***S4 Table:*** *Oligonucleotide used for overlapping PCRs to construct chimeric viruses.*

| **Viral backbone** | **Chimeric virus** | **Template** | **Fragment 1** | | **Fragment 2** | |
| --- | --- | --- | --- | --- | --- | --- |
|  |  |  | **Forward primer** | **Reverse primer** | **Forward primer** | **Reverse primer** |
| DENV2 | SLA ZIKV |  | 101 | 2817 | 2816 | 422 |
|  | SLA AROV |  | 101 | 3181 | 3182 | 422 |
|  | SLA SLEV (5’ AGA) |  | 101 | 3173 | 3174 | 422 |
|  | SLA ILHV (5’ AGA) |  | 101 | 3177 | 3178 | 422 |
|  | SLA ROCV (5’ AGA) |  | 101 | 3179 | 3180 | 422 |
|  | SLA WNV | ICRep DENV2 | 101 | 3169 | 3170 | 422 |
|  | SLA JEV (5’ AGA) |  | 101 | 3171 | 3172 | 422 |
|  | SLA USUV (5’ AGA) |  | 101 | 3248 | 3249 | 422 |
|  | SLA YFV |  | 101 | 3236 | 3237 | 422 |
|  | SLA TBEV |  | 101 | 2938 | 2939 | 422 |
|  | SLA POWV |  | 101 | 2940 | 2941 | 422 |
|  | SLA NHUV |  | 101 | 2942 | 2943 | 422 |
|  | SLA AeFV |  | 101 | 2944 | 2945 | 422 |
| ZIKV | SLA DENV2 | ICRep ZIKV | 101 | 2741 | 2740 | 2738 |
|  | SLA AROV |  | 101 | 2776 | 2777 | 2738 |
|  | SLA YFV |  | 101 | 2859 | 2858 | 2738 |
|  | SLA TBEV |  | 101 | 2861 | 2860 | 2738 |
|  | SLA POWV |  | 101 | 2743 | 2742 | 2738 |
|  | SLA NHUV |  | 101 | 2901 | 2900 | 2738 |
|  | SLA AeFV |  | 101 | 2899 | 2898 | 2738 |
|  | SSL POWV |  | 101 | 2751 | 2750 | 2738 |
|  | SSL OHFV |  | 101 | 2753 | 2752 | 2738 |
|  | SSL cISFV |  | 101 | 2755 | 2754 | 2738 |
|  | 3WJ+SSL TBEV |  | 101 | 3335 | 3336 | 2738 |
|  | SLA DENV2 | IC ZIKV | 101 | 2741 | 2740 | 2739 |
|  | SLA YFV |  | 101 | 2859 | 2858 | 2739 |
|  | SLA TBEV |  | 101 | 2861 | 2860 | 2739 |
|  | SLA NHUV |  | 101 | 2901 | 2900 | 2739 |
|  | SLA AeFV |  | 101 | 2899 | 2898 | 2739 |
