# Supplementary material for: The RNA promoter for pathogenic orthoflaviviruses replication is universal and serves as target for viral inhibition
Source: PLoS Pathog. 2026 May 18;22(5):e1014233. doi: 10.1371/journal.ppat.1014233 (PMC13211259; doi:10.1371/journal.ppat.1014233)
Supplement: S5 Table — (DOCX) [file ppat.1014233.s007.docx]

***S5 Table:*** *Primer sequences*

| **Oligo name** | **Sequence** |
| --- | --- |
| 101 | TCCAGACTTTACGAAACACG |
| 422 | GTGTGGTTCTCCGTTACGTGTGG |
| 2738 | GTTTTTCATCTCGAGTGGCCCAGG |
| 2739 | GCAATCAAGTCCTAGGCTTCCAAAACCC |
| 2740 | GTTAGTCTACGTGGACCGACAAAGACAGATTCTTTGAGGGAGCTAAGCTCAACGTAGTTCTAACAGTGGTTTTATTTTGGATTTGGAAACGAGAGTTTCTG |
| 2741 | AAAGAATCTGTCTTTGTCGGTCCACGTAGACTAACAACTTATAGTGAGTCGTATTAAACGCGTGAGCTC |
| 2742 | GTGTGTGTGGGCACTTTAGTCAGTGTCCGCGGCGTTCTTTTGAACGTGAGTGTGTTGAGAAAGGTTTTATTTTGGATTTGGAAACGAGAGTTTCTG |
| 2743 | CACTGACTAAAGTGCCCACACACACGTGCAAGAAAATCTTATAGTGAGTCGTATTAAACGCGTGAGCTC |
| 2750 | GCGACAGTTCGAGTTTGAGCGTTCTTTTGAACGTAGCAACAGTATCAACAGGTTTTATTTTGGATT |
| 2751 | ACGTTCAAAAGAACGCTCAAACTCGAACTGTCGCAGTCTG |
| 2752 | GGCAGGGTTGGTGGAAGGAATTGCCCGCATCAGCCAGTCGTAGCAACAGTATCAACAGGTTTTATTTTGGATT |
| 2753 | CAATTCCTTCCACCAACCCTGCCGCTCAAACTCGAACTGTCGCAGTCTG |
| 2754 | CAGACTGCGACAGTTCGAGTTTGACTAAATTTTAAAGCAACAGTATCAACAGGTTTTATTTTGGATT |
| 2755 | TTAAAATTTAGTCAAACTCGAACTGTCGCAGTCTG |
| 2762 | TTTTACGCGTTTAATACGACTCACTATAAGTTGTTGATCTGTGTGAATCAGACTGCGATAGTTCGAGTTTGAAGCGAAAGCTAG |
| 2763 | TTTTACGCGTTTAATACGACTCACTATAAGTTGTTGATCTGTGTGAATCAGACTGCGATCAGTCGAGTTTGAAGCGAAAGCTAGCAAC |
| 2776 | GTACGAACTGTCGCAATGGTCTCACGCAGAAGAAATACTTATAGTGAGTCGTATTAAACGCGTGAGCTC |
| 2777 | TATTTCTTCTGCGTGAGACCATTGCGACAGTTCGTACCGGTGAGTTTTGACTTAACGCAGTGAGAAAGGTTTTATTTTGGATTTGGAAACGAGAGTTTCTG |
| 2816 | AGTTGTTGATCTGTGTGAATCAGACTGCGACAGTTCGAGTTTGAAGCGAAAGCTAGCAACAGTATCAACATTTTTAATTAGAGAGCAGATCTCTGATGAATAAC |
| 2817 | CTGATTCACACAGATCAACAACTTATAGTGAGTCGTATTAAATTTACG |
| 2858 | AGTAAATCCTGTGTGCTAATTGAGGTGCATTGGTCTGCAAATCGAGTTGCTAGGCAATAAACACATTTGGATTAGGTTTTATTTTGGATTTGGAAACGAGAGTTTCTG |
| 2859 | CAATGCACCTCAATTAGCACACAGGATTTA**CT**TATAGTGAGTCGTATTAAACGCGTGAGCTC |
| 2860 | CGTTTGCTTCGGACAGCATTAGCAGCGGTTGGTTTGAAAGAGATATTCTTTTGTTTCTACCAGTCGTGAACGTGTTGAGAAAAAGGTTTTATTTTGGATTTGGAAACGAGAGTTTCTG |
| 2861 | CTGCTAATGCTGTCCGAAGCAAACGCATGCACGTGCAAGAAAAT**CT**TATAGTGAGTCGTATTAAACGCGTGAGCTC |
| 2898 | AGTTTTTAAAAACTTCTGTGAAGGTCACATCCCTAGTTGGATTACGCCGCTTTTGAAAAATCTAGAAGTTGTTTGGAAAAGGTTTTATTTTGGATTTGGAAACGAGAGTTTCTG |
| 2899 | GGGATGTGACCTTCACAGAAGTTTTTAAAAA**CT**TATAGTGAGTCGTATTAAACGCGTGAGCTC |
| 2900 | AGTTTGTGCTGTACGTGCACTGGAAGTACATAGTTGGTATAACGGGTAGAAATATAACGTATTCGCGCAAGGTTTTATTTTGGATTTGGAAACGAGAGTTTCTG |
| 2901 | CCAGTGCACGTACAGCACAAA**CT**TATAGTGAGTCGTATTAAACGCGTGAGCTC |
| 2938 | CGCTGCTAATGCTGTCCGAAGCAAACGCATGCACGTGCAAGAAAATCTTATAGTGAGTCGTATTAAATTTACGCGTGAG |
| 2939 | TTGCTTCGGACAGCATTAGCAGCGGTTGGTTTGAAAGAGATATTCTTTTGTTTCTACCAGTCGTGAACGTGTTGAGAAAATTTTTTAATTAGAGAGCAGATCTCTGATGAATAACC |
| 2940 | CGCACACACGTGCAAGAAAATCTTATAGTGAGTCGTATTAAATTTACGCGTGAG |
| 2941 | AGATTTTCTTGCACGTGTGTGCGGGTGCTTTAGTCAGTGTCCGCAGCGTTCTGTTGAACGTGAGTGTGTTGAGAAAATTTTTTAATTAGAGAGCAGATCTCTGATGAATAACC |
| 2942 | CCAGTGCACGTACAGCACAAACTTATAGTGAGTCGTATTAAATTTACGCGTGAG |
| 2943 | AGTTTGTGCTGTACGTGCACTGGAAGTACATAGTTGGTATAACGGGTAGAAATATAACGTATTCGCGCATTTTTTAATTAGAGAGCAGATCTCTGATGAATAACC |
| 2944 | GGATGTGACCTTCACAGAAGTTTTTAAAAACTTATAGTGAGTCGTATTAAATTTACGCGTGAG |
| 2945 | AGTTTTTAAAAACTTCTGTGAAGGTCACATCCCTAGTTGGATTACGCCGCTTTTGAAAAATCTAGAAGTTGTTTGGAAATTTTTTAATTAGAGAGCAGATCTCTGATGAATAACC |
| 2983 | TCGAGCTCACGCGTTTAATACGACTCACTATAAGAGTTTGTGCTGTACGTGCACTGGATGTACATAGTTG |
| 3124 | GAGCTCACGCGTTTAATACGACTCACTATAAGTTGTTGATCTGTGTGAATCAGACTGCGATGGTTCGAGTTTG |
| 3125 | GAGCTCACGCGTTTAATACGACTCACTATAAGTTGTTGATCTGTGTGAATCAGACTGCGACGGATCGAGTTTGAAG |
| 3128 | GAGCTCACGCGTTTAATACGACTCACTATAAGTTGTTGATCTGTGTGAATCAGACTGCGACAGTTCGAGTTTGAGTTGCTAGGCAATAGCAACAGTATC |
| 3169 | AGTTTGTCAGCTCACACAGGCGAACTACTTATAGTGAGTCGTATTAAATTTACGCGTGAG |
| 3170 | AGTAGTTCGCCTGTGTGAGCTGACAAACTTAGTAGTGTTTGTGAGGATTAACAACAATTAACACAGTGCGAGCTGTTTTTTAATTAGAGAGCAGATCTCTGATGAATAACC |
| 3171 | GAAGTTCACACAGATAAACTTCTTATAGTGAGTCGTATTAAATTTACGCGTGAG |
| 3172 | AGAAGTTTATCTGTGTGAACTTCTTGGCTTAGTATCGTTGAGAAGAATCGAGAGATTAGTGCAGTTTAAACTTTTTTAATTAGAGAGCAGATCTCTGATGAATAACC |
| 3173 | TGTTTCCTCTCCGCTCACCGACGCGAACATCTTATAGTGAGTCGTATTAAATTTACGCGTGAG |
| 3174 | GTCGGTGAGCGGAGAGGAAACAGATTTCCTTTTTGGAGGATAATAACTTAACTTGACTGCGAACATTTTTTAATTAGAGAGCAGATCTCTGATGAATAACC |
| 3177 | ACGGTTAGTGAAATTCACACAGGTGAATTTCTTATAGTGAGTCGTATTAAATTTACGCGTGAGCTC |
| 3178 | ACCTGTGTGAATTTCACTAACCGTTTTAGTGGAGAGAACTTTTGTTTAACACAGTCTGAATTTTTTTAATTAGAGAGCAGATCTCTGATGAATAACC |
| 3179 | AAAACTGTTGGTGAAATTTCACACAGGTGAATTTCTTATAGTGAGTCGTATTAAATTTACGCGTG |
| 3180 | TTCACCTGTGTGAAATTTCACCAACAGTTTTAGTGAAAAGAACTTTGTTTAACACAGTCTGAATTTTTTTAATTAGAGAGCAGATCTCTGATGAATAACC |
| 3181 | GTCGCAATGGTCTCACGCAGAAGAAATACTTATAGTGAGTCGTATTAAATTTACGCGTGAG |
| 3182 | CTTCTGCGTGAGACCATTGCGACAGTTCGTACCGGTGAGTTTTGACTTAACGCAGTGAGAAATTTTTTAATTAGAGAGCAGATCTCTGATGAATAACC |
| 3236 | TCTGCTCTCTAATTAAAAAATTAATCCAAATGTGTTTATTG |
| 3237 | AACACATTTGGATTAATTTTTTAATTAGAGAGCAG |
| 3247 | CTGTTTCCTCTCCGCTCACCGACGCGAACAACTTATAGTGAGTCGTATTAAATTTACGCGTGAG |
| 3248 | AACAATACTAAGTAGTAGAGCTCACACAGGCCAACATCTTATAGTGAGTCGTATTAAATTTACGCGTGAG |
| 3249 | GCCTGTGTGAGCTCTACTACTTAGTATTGTTTTTGGAGGATCGTGAGATTAACACAGTGCCGGCAGTTTTTTAATTAGAGAGCAGATCTCTGATGAATAAC |
| 3332 | CGAGCTCACGCGTTTAATACGACTCACTATAAGTTGTTGATCTGTGTGCATCAGACTGCGACAGTTCGAGTTTGAAGCGAAAGCTGAACAACAGTATC |
| 3335 | CAAACCAACCGCTCAAACTCGAACTGTCGCAGTCTGATGCACACAGATCAACAACTTATAGTGAG |
| 3336 | TTCGAGTTTGAGCGGTTGGTTTGAAAGAGATATTCTTTTGTTTCTACCAGTCGTGAACAACAGTATCAACAGGTTTTATTTTGGATTTGG |
| 3339 | TTCGAGCTCACGCGTAAATTTAATACGACTCACTATAAGTAGTTTATC |
| 3340 | TTCGAGCTCACGCGTAAATTTAATACGACTCACTATAAGTAATTCACC |
| 3341 | TTCGAGCTCACGCGTAAATTTAATACGACTCACTATAAGTTGTTGG |
| 3436 | AAAAAAACGCGTTTAATACGACTCACTATAAGTTGTTGATCTGTGTCTAATCAGACTGCGACAGTTCGAGTTTGAAGCGAAAGCTAAAGCAACAGTATCAACA |
