# Supplementary material for: The RNA promoter for pathogenic orthoflaviviruses replication is universal and serves as target for viral inhibition
Source: PLoS Pathog. 2026 May 18;22(5):e1014233. doi: 10.1371/journal.ppat.1014233 (PMC13211259; doi:10.1371/journal.ppat.1014233)
Supplement: S6 Table — (DOCX) [file ppat.1014233.s008.docx]

***S6 Table:*** *Primer sequences to construct SLA RNAs for in vitro binding assays*

| **RNA** | **Template** | **Forward primer sequence** | **Reverse primer sequence** |
| --- | --- | --- | --- |
| SLA DENV2 | ZIKV with SLA DENV2 | 5′TCGTTAATACGACTCACTATAGGAGTTGTTAGTC3’ | 5′CTCCGCGTTTTAGCATATTGACAATC3’ |
| SLA TBEV | ZIKV with SLA TBEV | 5′GCGTTTAATACGACTCACTATAGGAGATTTTC3’ | 5′CTCCGCGTTTTAGCATATTGACAATC3’ |
| SLA YFV | ZIKV with SLA YFV | 5′GCGTTTAATACGACTCACTATAGGAGTAAATC3’ | 5′CTCCGCGTTTTAGCATATTGACAATC3’ |
| SLA AEFV | ZIKV with SLA AeFV | 5′GCGTTTAATACGACTCACTATAGGAGTTTTTAAAAAC3’ | 5′CTCCGCGTTTTAGCATATTGACAATC3’ |
| SLA NHUV | ZIKV with SLA NHUV | 5′GCGTTTAATACGACTCACTATAGGAGTTTG3’ | 5′CTCCGCGTTTTAGCATATTGACAATC3’ |
| Non-related RNA | ZIKVRep | 5′TAATACGACTCACTATAGGCCATGATTGGGGTGC3’ | 5′CTTCTTCAGATTTGATCAACGCAATATCTTCTTC3’ |
